# Loss of perivascular aquaporin-4 localization impairs glymphatic exchange and promotes amyloid β plaque formation in mice

**DOI:** 10.1186/s13195-022-00999-5

**Published:** 2022-04-26

**Authors:** Matthew Simon, Marie Xun Wang, Ozama Ismail, Molly Braun, Abigail G. Schindler, Jesica Reemmer, Zhongya Wang, Mariya A. Haveliwala, Ryan P. O’Boyle, Warren Y. Han, Natalie Roese, Marjorie Grafe, Randall Woltjer, Detlev Boison, Jeffrey J. Iliff

**Affiliations:** 1 Neuroscience Graduate Program, Oregon Health & Science University, Portland, OR USA; 2 Department of Anesthesiology and Perioperative Medicine, Oregon Health & Science University, Portland, OR USA; 3 Department of Psychiatry and Behavioral Sciences, University of Washington School of Medicine, Seattle, WA USA; 4 Center for Advanced Biomedical Imaging, University College London, London, UK; 5 VISN 20 Mental Illness Research, Education and Clinical Center (MIRECC), VA Puget Sound Health Care System, 1660 S Columbian Wy., Seattle, WA 98108 USA; 6 VISN 20 Geriatric Research, Education and Clinical Center (GRECC), VA Puget Sound Health Care System, Seattle, WA USA; 7 Robert Stone Dow Neurobiology Laboratories, Legacy Research Institute, Portland, OR USA; 8 Department of Pathology, Oregon Health & Science University, Portland, OR USA; 9 Department of Neurosurgery, Robert Wood Johnson Medical School, Rutgers University, Piscataway, NJ USA; 10 Knight Cardiovascular Institute, Oregon Health & Science University, Portland, OR USA; 11 Department of Neurology, University of Washington School of Medicine, Seattle, WA USA

**Keywords:** Alzheimer’s disease, Aquaporin-4, AQP4, α-Syntrophin, glymphatic, Amyloid β, Perivascular, Astrocyte

## Abstract

**Background:**

Slowed clearance of amyloid β (Aβ) is believed to underlie the development of Aβ plaques that characterize Alzheimer’s disease (AD). Aβ is cleared in part by the glymphatic system, a brain-wide network of perivascular pathways that supports the exchange of cerebrospinal and brain interstitial fluid. Glymphatic clearance, or perivascular CSF-interstitial fluid exchange, is dependent on the astroglial water channel aquaporin-4 (AQP4) as deletion of *Aqp4* in mice slows perivascular exchange, impairs Aβ clearance, and promotes Aβ plaque formation.

**Methods:**

To define the role of AQP4 in human AD, we evaluated AQP4 expression and localization in a human post mortem case series. We then used the α-syntrophin (*Snta1*) knockout mouse model which lacks perivascular AQP4 localization to evaluate the effect that loss of perivascular AQP4 localization has on glymphatic CSF tracer distribution. Lastly, we crossed this line into a mouse model of amyloidosis (Tg2576 mice) to evaluate the effect of AQP4 localization on amyloid β levels.

**Results:**

In the post mortem case series, we observed that the perivascular localization of AQP4 is reduced in frontal cortical gray matter of subjects with AD compared to cognitively intact subjects. This decline in perivascular AQP4 localization was associated with increasing Aβ and neurofibrillary pathological burden, and with cognitive decline prior to dementia onset. In rodent studies, *Snta1* gene deletion slowed CSF tracer influx and interstitial tracer efflux from the mouse brain and increased amyloid β levels.

**Conclusions:**

These findings suggest that the loss of perivascular AQP4 localization may contribute to the development of AD pathology in human populations.

**Supplementary Information:**

The online version contains supplementary material available at 10.1186/s13195-022-00999-5.

## Background

The aberrant accumulation of soluble proteins into insoluble aggregates is the central histopathological hallmark of a broad range of neurodegenerative diseases including Alzheimer’s disease (AD), Huntington’s disease, amyotrophic lateral sclerosis, and Parkinson’s disease. In each, this pathology develops with advancing age and often follows a stereotyped neuroanatomical distribution that parallels clinical disease progression. Alzheimer’s is characterized histopathologically by the formation of extracellular plaques made up of amyloid β (Aβ) and intracellular neurofibrillary tangles made up of hyper-phosphorylated species of the microtubule-associated protein tau (p-tau) [1, 2]. Yet the changes that occur in the aging brain that render it vulnerable to the development and progression of these aggregates remain unknown.

Pulse-chase kinetic studies in humans demonstrate that the clearance of Aβ is slowed both with advancing age [3] and in the most common sporadic form of AD [4] while in these settings the production of Aβ remains largely unchanged. This suggests that impairment of processes supporting the clearance of Aβ may drive the development of amyloid pathology. Interstitial Aβ is cleared locally through cellular uptake and degradation [5–8], efflux across the blood-brain barrier [9, 10], and through clearance by the glymphatic system which supports exchange to the cerebrospinal fluid (CSF) compartment along perivascular pathways [11, 12]. This perivascular CSF-interstitial fluid (ISF) exchange is impaired in rodent models of aging [13], traumatic brain injury [14], and cerebral small vessel disease [15]. Each of these is an established AD risk factor in humans, suggesting that impaired perivascular exchange may be an important contributor to the development of Alzheimer’s pathology and disease progression [16]. Yet the mechanism underlying age-related impairment of this perivascular clearance pathway and its potential role in the development of AD in humans remains undefined.

Glymphatic exchange is dependent upon the astroglial water channel aquaporin-4 (AQP4), which localizes to the perivascular astrocytic endfeet ensheathing the brain vasculature [11, 17]. In mice, deletion of *Aqp4* slows perivascular CSF influx into brain tissue, slows interstitial Aβ clearance, and accelerates Aβ plaque formation [11, 17, 18]. Consistent with findings in aged mice [13] and in mouse models of AD [19], in a preliminary study of post mortem human frontal cortical tissue we observed that subjects with a clinical AD diagnosis exhibited changes in astroglial AQP4 localization, including the loss of AQP4 from perivascular endfeet and its distribution to non-perivascular processes [20]. To confirm these findings and extend them along the clinico-pathological spectrum of AD, here we evaluate cortical and hippocampal AQP4 localization in a more extensive human post mortem case series including subjects >65 years of age that were cognitively intact and had clinical diagnoses of mild cognitive impairment (MCI) or of AD. We observe that reduced frontal cortical perivascular AQP4 localization is associated with local Aβ and p-tau pathology, as well as with cognitive decline early in the course of clinical AD progression.

Although changes in perivascular AQP4 localization in mouse models of aging [13], traumatic brain injury [14, 21], and cerebrovascular injury [15, 22] were each associated with slowed glymphatic function, whether changes in perivascular AQP4 localization impair CSF-ISF exchange and contribute to the development of Alzheimer’s pathology has not yet been directly tested. Here we comprehensively define changes in perivascular AQP4 localization in the aging rodent brain along both the microvasculature and large penetrating vessels. We observe two co-occurring phenotypic changes in AQP4 localization: (1) the loss of AQP4 from perivascular endfeet along the brain microcirculation, and (2) the distribution of AQP4 to non-perivascular fine astroglial processes, particularly along large penetrating and intraparenchymal vessels.

Lastly, we use two distinct experimental approaches to define whether either of these phenotypic changes slow perivascular solute exchange or promote the development of AD-related Aβ pathology. Using an adeno-associated virus (AAV)-based approach to drive astrocyte-specific AQP4 overexpression, we recapitulate the distribution of AQP4 to astroglial fine processes and observe that this change does not impair glymphatic function or alter Aβ levels in the Tg2576 mouse model of amyloidosis [23]. We then utilize a genetic model in which the gene encoding α-syntrophin (*Snta1*), which anchors AQP4 at the perivascular endfoot, is deleted to model the loss of perivascular AQP4 localization. In these mice, the loss of AQP4 localization impairs perivascular exchange and increased Aβ levels in the Tg2576 line.

## Methods

### Human post mortem histopathological study

#### Subjects

Human samples and subject clinical and neuropathological information including age, sex, Braak stage for tau pathology, Consortium to Establish a Registry for Alzheimer’s Disease (CERAD) score for neuritic plaques, clinical-pathological diagnosis, and measures of global cognitive and functional status were obtained from the Oregon Health and Science University (OHSU) Layton Aging and Alzheimer’s Disease Center and associated post mortem tissue repository, the Oregon Brain Bank. Methods used to generate clinical and pathological data have been previously described [24]. All tissue came from volunteers that already signed written informed consent to allow future use of their data. All subjects underwent brain autopsy after consent was obtained from the next of kin and in accordance with the OHSU guidelines. The research protocols for these studies were reviewed and granted ethical approval by the OHSU Institutional Review Board. Inclusion criteria for the study were (a) availability of both fixed and frozen brain tissue within the Oregon Brain Brank, (b) age 65 or older, (c) a primary neuropathological diagnosis of definite AD, probable AD or possible AD using CERAD criteria, or Normal Brain; (d) a secondary neuropathological diagnosis of either “No secondary neuropath diagnosis” or “AD pathology present but insufficient for AD diagnosis.” Exclusion criteria included a primary or secondary neuropathological diagnosis other than those listed above. These criteria were intended to identify tissues featuring a spectrum of AD-related Aβ and p-tau pathology, but without other significant neuropathology, such as cerebrovascular disease. A total of 76 subjects, both male and female, with an age range of 75–105 were included in this study. For the frontal cortex analysis, 53 of the immunostained sections were successfully stained, imaged, and analyzed. For the hippocampal tissue analysis, 36 of the immunostained sections were successfully stained, imaged, and analyzed.

#### Immunofluorescence

Following post mortem processing, brains were fixed for at least 2 weeks in 10% neutral buffered formalin and dissected into regions of interest, and then embedded into paraffin blocks. Sections were sliced on a microtome at 7-μm thickness. To minimize the impact of staining protocol variability, cortical and hippocampal samples from all subjects were labeled in two parallel bathes. Tissue sections, selected from the hippocampus and the frontal cortex, were deparaffinized and treated with 10% formic acid. Antigen retrieval was performed in sodium citrate buffer (pH 6.0) in a steamer for 20 min. Sections were incubated with 3% normal donkey serum in phosphate-buffered saline with 1% BSA and 0.1% Triton (blocking serum) for 20 min at room temperature. Samples were incubated in the primary antibody diluted in blocking serum overnight at 4 °C. Primary antibodies used were as follows: rabbit anti-AQP4 (1:800; Millipore; catalog #AB3594), mouse anti-human phospho-PHF-Tau monoclonal (AT8; 1:1000; Thermo Fisher; catalog #MN1020). Following overnight primary incubation, sections were incubated with secondary antibody for 1 h at room temperature. Secondary antibodies used were donkey anti-rabbit Alexa Fluor 488 (1:400; Life Technologies, catalog #A21207) and donkey anti-mouse Alexa Fluor 594 (1:400; Life Technologies; catalog #A21202). Slices were mounted using Fluoromount-G mounting medium containing Hoechst 33342 nuclear stain (1:10000; Molecular Probes; catalog #H3570). IF imaging was performed on the Zeiss Axio Scan.Z1 fluorescent scanner. In a prior study from the Oregon Brain Bank, we observed no significant association between post mortem interval and AQP4 immunofluorescence (IF) or localization in fixed frontal cortical tissue [20].

#### Quantification of local amyloid β and P-tau pathology in human tissue

Micrographs were analyzed using FIJI ImageJ software (version 1.52i) and the investigator was blinded to subject dementia status. For the cortex, 12 ROIs that were 1000 × 1000 pixels were placed across the section, with 6 spread across the frontal cortex grey matter and 6 across the frontal cortex white matter. Composite micrographs were then separated into their single channel images for analysis. Each ROI was then extracted and a grid placed across it for ease of counting. P-tau-positive cells were counted using the manual cell counter function and included as a total count per ROI. The ROI was then converted into square millimeter area and the total counts were converted to calculate counts per square millimeter. These were averaged across the 6 ROIs to give each subject a single count value. For the hippocampus, ROIs were drawn in the CA1, CA2, and CA3 regions by an experienced neuropathologist. Due to section availability, orientation of cutting, and section quality, only a subset of hippocampal sections were analyzed. P-tau labeling was tallied using the manual cell counter function and included as a total count per ROI. Each ROI was then converted into square millimeter area and the plaque counts converted to present them as counts per square millimeter for CA1, CA2, and CA3 regions separately.

#### Analysis of AQP4 immunofluorescence in human tissue

High levels of autofluorescence and lipofuscin staining present in the post mortem human tissue generally, and the pathological tissue (MCI and AD) specifically, rendered the type of granular line-based analysis that we employed in rodent tissue (see below) impracticable. For this reason, we adopted a coarser threshold-based approach similar to those we have employed in prior studies [13, 20]. A schematic detailing the analysis workflow is provided in Supplemental Figure 1. Composite micrographs were separated into their respective channels, and ROIs used in the p-tau analysis opened on the red channel. ROIs were occasionally moved in instances where the red channel section was out of focus. Each ROI was duplicated and the mean unthresholded intensity and area was measured. A threshold was then applied to highlight the edges of vessels, which was designated as the vessel threshold, and the minimum value at this threshold recorded. A second threshold was then extended to capture the cellular threshold including the intensity at the fine processes of astrocytes where they could be readily identified. This was designated the cellular threshold and the mean value and area of this threshold was recorded. Area coverage and AQP4 polarization ratio was calculated. The average of 6 ROIs were taken for the frontal cortical gray and white matter per subject. Single values were obtained per subject for the hippocampal CA1, CA2, and CA3 regions.

### Rodent study of perivascular AQP4 localization

#### Animals

C57Bl6/J mice were obtained from the Jackson Laboratories. Homozygous B6;SJL-Tg(APPSWE)2576Kha mice were generated by Dr. Karen Hsiao [23] and were obtained from Taconic (stock no. 1349), before being crossed onto and maintained on a C57Bl6/J background. Homozygous *Stna1*^−/−^ mice were generated by Dr. Stanley Froehner [25] and were obtained from Jackson Laboratories (stock no. 012940). *Stna1*^−/−^ mice were maintained on a C57Bl6/J background and age-matched C57Bl6/J mice obtained from Jackson Laboratories were used as wild type controls for *Stna1*^−/−^ homozygous. Tg2576(+);*Stna1*^−/−^ mice and their littermate controls, Tg2576(-);*Stna1*^+/*+*^ and Tg2576(+);*Stna1*^+/*+*^ mice, were generated by first crossing Tg2576(+) with *Stna1*^+/−^ then crossing Tg2576(+);*Stna1*^+/−^ mice with each other.

All mice were cared for by the Oregon Health & Science Department of Comparative Medicine in an Association for Assessment and Accreditation of Laboratory Animal Care (AALAC) accredited vivarium. All experiments were performed in accordance with state and federal guidelines and all experimental protocols were approved by the institutional animal care and use committee (IACUC).

#### Materials and antibodies for rodent histological studies

In total, 10 kD Cascade Blue-conjugated dextran (Thermo Fisher D1976) and 70 kD Texas Red-conjugated dextran (Thermo Fisher D1864) were dissolved in saline. Aliquots of a 1:1 combined mixture of the dextrans were made and frozen at – 20 °C until used. Then, 70 kD Texas Red-conjugated dextran was also used as an intravascular tracer for in vivo 2-photon experiments. Taqman assays used to measure gene expression include human AQP4 pan-isoform (Hs0024342_m1, Life Technologies) and mouse β-actin (Mm00607939_s1, Life Technologies). Primary antibodies included rabbit anti-AQP4 (1:500, Millipore AB3594) and mouse anti-β amyloid (1:200, Novus Biologicals NBP2-13075, mouse-human cross-reactive). Conjugated lectin-DyLight® 594 label (VECTOR LABORATORIES DL-1177-1) was added at 1:100 dilution when needed. All secondary antibodies were generated in donkey and conjugated to either Alexafluor-488, Alexafluor 594, or Alexafluor 647. All secondary antibodies for IF were used at a concentration of 1:500 and were ordered from Thermo Fisher. Hoescht 33342 (1:5000, Thermo Fisher H3570) was used to label cell nuclei. Human AQP4 used in viral studies was derived from a human AQP4 cDNA plasmid (Sino HG15306-G). Parent plasmid used for viral constructs was generated and deposited to Addgene by Bryan Roth (Addgene plasmid #50478; http://n2t.net/addgene:50478 ; RRID:Addgene_50478). HA-tagged AQP4 viruses were generated by Vector Biolabs. Quantification of amyloid β was performed using commercially available human-specific enzyme-linked immunosorbent assay (ELISA) detection kits for amyloid β_1-40_ or amyloid β_1-42_ (Fisher Scientific KHB3481 and KHB3441 respectively).

#### Virus production

All AAV constructs utilized in this study are detailed in Supplementary Table 1. Recombinant AAV expression plasmids were generated by adapting the parent pAAV-GfaABC1D plasmid (Roth lab, Addgene plasmid #50478) AAV constructs contained 3′ woodchuck hepatitis virus posttranscriptional regulatory element (WPRE) to promote gene expression and stability of intronless RNA [26]. Recombinant AAV8 and PHP.B were packaged in cultures of HEK 293T cells. Approximately 1.5 × 107 293T cells were seeded into 150-mm dishes in complete DMEM supplemented with 10% fetal bovine serum, 1 mM MEM sodium pyruvate, and 1% Penicillin-Streptomycin (100 units/mL) and grown at 37 °C, 5% CO_2_. At 24 h, media was replaced with fresh culture media and, after 2 h, cells were then transfected with three separate plasmids: (1) Adeno helper plasmid (pFΔ6), (2) AAV helper encoding the Rep 2 and Cap 8 sequences for serotype 8 (pAR8) or PHP.B [27], and (3) the recombinant AQP4 plasmids described above. Cells were harvested between 48 h and 72 h post transfection by scraping and viruses crudely isolated from cell membranes via freeze-thaw cycling and treatment with benzonase (50 U/mL). This crude lysate was clarified by centrifugation, then the supernatant was purified via centrifugation of a discontinuous iodixanol density gradient [28]. The purified virus was then buffer exchanged and concentrated in sterile DPBS by centrifugation in Amicon Ultra-15 Centrifugal Filter Units. The final preparation was sterile filtered through a syringe filter. Viral titers were determined by quantitative RT-PCR using primers specific to the WPRE sequence.

#### Primary astrocyte cultures

Primary astrocyte cultures were generated using a serum-containing-media-based approach derived from classic astrocyte culture preparation protocol, rather than an immunopanning-based approach [29]. Briefly, cortical homogenates were collected on ice from neonatal pups aged P2-P5. Astrocytes were purified over the course of several centrifugation and filtering steps. Astrocyte solutions were plated at an initial density of ~100,000 cells/cm^2^ in a 75-cm^2^ flask, grown to ~60% confluency prior to plating on Poly-D-Lysine-coated coverslips. While on glass coverslips, viral treatments were administered in 10% FBS-containing media over the course of several days prior to imaging experiments. Cells were fixed with 4% paraformaldehyde at room temperature prior to mounting, staining, and imaging.

#### Anesthesia

For all acute time-course experiments, anesthesia was briefly induced under isoflurane (2–4%), then maintained under ketamine/xylazine (KX) anesthesia for the duration of the experiment (0.10 mg/g ketamine, 0.01 mg/g xylazine, intraperitoneal). During all surgeries, mouse body temperature was maintained by heating pads and overhead heating lamps. For long surgeries (90–120 min), mice were given intraperitoneal saline approximately halfway through the surgical procedure to minimize dehydration.

#### Thin skull surgery and in vivo 2-photon imaging

Viral constructs were delivered via intraparenchymal injection approximately 4 weeks prior to imaging experiments. On the day of imaging, a thin skull preparation was performed above the motor cortex. Briefly, mice were anesthetized under isoflurane and head-fixed in a three-point stereotax. Skin was deflected to expose the skull, then a 10% ferric chloride solution was used to dry the skull and allow for removal of overlying tissues. A head plate was then fixed on the skull with superglue, centered over the right motor cortex. A microtorque drill was used to gently thin the skull down through the diploe, across a 3-mm diameter region. During this process, saline was intermittently applied to the surface of the skull to minimize heat damage at the surface of the brain. A dental microblade was then used to further thin the skull to a uniform thickness of under 50 μm and a glass coverslip was superglued in place over the thinned skull. To image the vasculature, a blood-brain barrier-impermeable Texas red-conjugated 70 kD dextran was injected into the vascular system via the right retro-orbital sinus. Mice were then slowly transitioned to KX anesthesia over the course of 15 min and moved in the stereotaxic frame to a Zeiss LSM 7MP multiphoton microscope equipped with dual channel binary GaAsP (BiG) detectors and a Coherent Technologies Chameleon titanium-sapphire femtosecond pulsed laser source. Two-photon images were acquired using a Zeiss LSM 7MP intravital 2-photon microscope with a W Plan-apochromatic ×20 (1.0DIC D=0.17 M27 75mm) objective. Green and red channel fluorescence were simultaneously acquired using the GaAsP NDD detector. Sixteen-bit 1024 × 1024 images were acquired (pixel size: 0.42 × 0.42 × 0.72 μm) with unidirectional scanning and a pixel dwell time of 0.79 μs.

#### Real-time PCR

Total RNA was extracted from brain homogenate using a mirVana Paris RNA purification kit. A cDNA library was then constructed using a high-capacity reverse transcription kit (Applied Biosystems). Taqman assays were then performed using a thermal cycler according to manufacturer recommendations.

#### Protein isolation and Western blot

Whole-brain tissue lysates were generated by dounce homogenization in RIPA buffer (Thermo Fisher 89900) with added protease inhibitor cocktail (Sigma-Aldrich 11836170001). Samples were spun at 2000*g* for 10 min at 4 °C to remove cellular debris then frozen at – 80 °C. Protein concentration was quantified by Pierce BCA protein assays (Thermo Fisher 23225). Western blots were run on NuPage 4–12% Bis-tris gels. Fifty micrograms of protein was combined with LDS sample buffer and run on the gel for 90 min at 200 mV on ice at 4 °C. Protein transfer was done using Immobilon-FL membranes and was run at 30 mV for 60 min at 4 °C. Primary and secondary antibodies were applied over the course of 3 h using the Invitrogen iBind Flex system. Bands were imaged using a Licor Odyssey CLx fluorescence gel imaging system. Values for each sample were generated by averaging across two independent blots. Abundance was determined by measuring the abundance relative to the loading control band (β-actin).

#### Amyloid β soluble and insoluble fraction extraction

Frozen forebrain tissues were homogenized and sonicated on ice at 0.1 g/mL in cold tris-buffered saline solution that contained 1% triton X-100, 5 mM EDTA, protease inhibitor (Roch Cat#11836170001), and phosphatase inhibitor (Thermo Fisher Cat#88667). The homogenates were then centrifuged at 100,000 rpm for 1 hour at 4 °C. The supernatants were collected and used for the measuring the soluble fraction of Aβ by ELISA. The pellets were resuspended in the same homogenization buffer, sonicated on ice, and centrifuged at 100,000 rpm for 1 h at 4 °C. This step was repeated twice to wash off all soluble amyloid β fractions. The pellets were then suspended in deionized water, sonicated on ice, and centrifuged at 100,000 rpm for 20 min at 4 °C to wash off triton x-100. The pellets were then resuspended in 70% formic acid, sonicated on ice, and centrifuged at 100,000 rpm for 1 h at 4 °C to extract insoluble amyloid β fraction. Next, the supernatants were collected and dried out in SpeedVac Vacuum Concentrator (Thermo Fisher). Finally, the films were resuspended in 5 M guanidinium chloride with 100 mM tris (pH 7.4–8.5) and used for measuring the insoluble amyloid β fraction. To avoid guanidinium chloride interfering with antibody binding, the concentrated insoluble amyloid β fraction was diluted at least 50 times before performing ELISA.

#### Intravenous AAV injection

Twenty-five-gauge needles were backfilled with saline, then loaded with the appropriate volume of AAV in aCSF to achieve target viral concentration (typically 1 × 10^11^ vg). Mice were anesthetized with isoflurane and head position was stabilized with a tooth bar. To improve access to the retro-orbital compartment, gentle pressure was applied around the eye. The needle was then inserted, bevel down at the medial canthus. Following the orbital bone, the needle was shifted to access the retro-orbital sinus. Virus was then slowly injected into the retro-orbital compartment and the needle was withdrawn.

#### Intracisternal tracer infusion

Tracer infusions were performed as previously described [11, 17]. Briefly, a 30-gauge needle tip was connected to a 10-μl Hamilton syringe (Hamilton 80001) by PE-10 tubing. The line was filled with saline and tracer cocktails were backfilled immediately prior to injections. Mice were lightly anesthetized with isoflurane to minimize stress, then KX anesthesia was administered intraperitoneally. Mice were head-fixed in a stereotax and the skin of the neck was exposed with Nair. An incision was made along the midline of neck muscular layers and the tissue was deflected to expose the atlanto-occipital membrane covering the cisterna magna. The needle was inserted into the cisterna magna compartment, until the bottom of the bevel had passed through the atlanto-occipital membrane, then VetBond was used to secure the needle in place. A syringe pump was used to drive tracer into the cisterna magna at a rate of 500 nl/min, for 8 min, resulting in the infusion of 4 μl total. Successful injection was confirmed immediately, by visualization of tracer pulsation within the cisterna magna, and after tissue collection by observing tracer along perivascular spaces. Mice were maintained under KX anesthesia for the duration of the 90-min experiments, with intraperitoneal redosing administered as necessary. Depth of anesthesia was monitored by pedal reflex throughout the course of the experiment. Body temperature was maintained between 36 and 38 °C using a heating pad and lamp. Upon experiment completion, brain tissue was fixed with transcardiac perfusion of 4% paraformaldehyde. Brain surfaces were imaged immediately following the experiment using a fluorescence dissection scope (Zeiss AxioImager upright microscope). Brains were then post-fixed in 4% paraformaldehyde overnight, followed by cryopreservation in 30% sucrose overnight, prior to freezing in OCT and stored at – 80 °C until sectioning.

#### Intraparenchymal injections

Intracortical injections targeted the motor cortex (1, −1.5, .3 mm relative to bregma). Intrastriatal injections targeted caudate putamen (2.8, −0.5, 3.5 mm relative to bregma). Anesthetized mice were head-fixed in a stereotaxic frame. Fur was cleared from the skin above the skull using Nair. An incision was made along midline to expose the skull and connective tissue was removed with a cotton-tipped applicator. A stereotax-mounted dremel drill was used to create a small burr hole at the medial/lateral and anterior/posterior bregma coordinates. The dremel was replaced with a 10-μl Hamilton syringe outfitted with a 32-gauge needle (Hamilton 80014) that was loaded into a syringe pump and backfilled with fluorescently conjugated dextran. The needle tip was then lowered slowly into the brain through the burr hole to the depth of the dorsal/ventral bregma coordinates. The needle was left in place for 5 min prior to the start of infusion. Infusions were performed at a rate of 50 nl/min for 20 min for a final volume of 1 μl to minimize pressure-driven bulk flow of tracer. The needle was left in place and anesthesia depth was monitored and adjusted as necessary (as described above) over the course of the experimental time frame (120 min). Upon completion of both experiments, transcardiac paraformaldehyde perfusion was performed under anesthesia. Brain surfaces were imaged to confirm successful infusion and minimal recirculation of tracer via the CSF (indicating the needle tip was in the CSF or ventricle) when fluorescent tracers were injected. Brains were post-fixed overnight in paraformaldehyde, followed by an overnight incubation in 30% sucrose to cryo-protect the tissue. Brains were then frozen in OCT compound and stored at – 80 °C until sectioning.

#### Tissue preparation and immunostaining

Brains were serially sectioned on cryostat at a thickness of 20–50 μm optimized for individual staining. All IF staining was performed on free floating sections. Sections were blocked in phosphate-buffered saline solution containing 0.3% triton x-100, 5% normal donkey serum, and 2% bovine serum albumin (BSA) (optional) at room temperature. Primary and secondary antibodies were diluted in blocking solution and were incubated with the sections on a rocker overnight at 4 °C. When used, Hoescht 33342 was added into the secondary antibody solution or added in the last 10 min of washing. Tissue sections were mounted directly onto coverslips with a paintbrush and mounted with Fluoromount-G mounting media for all experiments with the exception of the hemagglutinin-tagged experiments for which Prolong-gold mounting media was used.

#### Slide scanning and confocal imaging

Full brain images of tracer distribution were acquired using automated, slide-scanning microscopy (Zeiss AxioScan.Z1) with a ×20 0.8 PlanApo objective. For AQP4 quantification along various vessel diameters, images were acquired on confocal microscopy (Nikon A1R) with a ×20 objective. High-magnification images were acquired using a laser scanning confocal microscope, equipped with an AiryScan super-resolution detector (Zeiss LSM880 with Fast Airyscan). Here, images were acquired at ×63 (1.4 PlanApo objective) or ×20 (PlanApo objective). To maximize the uniformity of quantifications across images, all laser intensities were selected to minimize signal outside of the dynamic range of the detector across all samples imaged.

#### Image and statistical analysis

All image analysis was performed using FIJI (ImageJ) [30] and IMARIS software. All statistical analysis was performed using GraphPad Prism 8. Uniform, linear adjustments to pixel intensities have been applied to images in figures presented here, but all analyses were run on images as acquired, or uniformly size-scaled images (due to computational memory constraints). IMARIS was used to render three-dimensional green fluorescent protein puncta (GFP volumes in z-stack images of cultured cells) for characterization of puncta size and number. Analyses including total brain and sub-regional brain tracer influx, and perivascular AQP4 localization were quantified in FIJI. Briefly, identification of subjective values including regions of interest (ROIs) and uniform threshold selection were performed in a blinded, manual fashion. After these values were selected, custom scripts were generated to automate analysis. Analyses of AQP4 expression along different cellular compartments was also described in detail in “Results” and Supplemental Figure 1 (human tissue) and Supplemental Figures 2 & 3 (mouse tissue) and performed by blinded quantifier. Capillaries were defined as vessels that were <10 microns in diameter; large vessels were >10 microns.

One-way or two-way analysis of variance (ANOVAs) were utilized to identify statistical differences in studies involving two or more groups with one to a few features. Mixed effects analyses were used when values were missing or to control for correlation within subject. *P* values were adjusted for multiple comparisons. For comparisons of two groups, unpaired *t*-test were used. All statistical tests are also listed in the text and figure legends. To examine associations between AQP4 localization and cognitive or pathological outcomes, we used linear regression and visualized associations with partial regression plots. All plots in figures are presented with mean. Due to the use of automated image acquisition software, occasionally a small percentage of images would exhibit poor focusing and were subsequently excluded from analysis (1–2 images/dataset). In these situations, integrated density was calculated across the remaining sections.

## Results

### Loss of perivascular AQP4 localization is associated with clinical diagnosis of Alzheimer’s disease and Alzheimer’s neuropathology in human post mortem samples

We first sought to characterize the changes in AQP4 localization associated with clinical AD diagnosis, Alzheimer’s pathology, and measures of global cognitive and functional status in a post mortem case series from the Oregon Brain Bank. In contrast with our preliminary study that examined the effect of age and cognitive status on AQP4 expression and localization in human frontal cortical tissue [20], in the present study, we sought a detailed comparison between AQP4 localization and Alzheimer’s pathology across the AD clinico-pathological spectrum in old age. Thus, we selected only older subjects (>65 years old at death) that exhibited primarily Alzheimer’s neuropathology, excluding those with histopathological evidence of other processes such as vascular disease, Lewy body disease, and hippocampal sclerosis. The case series included frontal cortical tissues from 53 subjects, and hippocampal tissue from 36 subjects. This included subjects with a premortem clinical diagnosis of cognitively normal (CN), mild cognitive impairment (MCI), and AD. Demographic information (including sex, *APOE4* allele status, education), cognitive and functional status (Mini-Mental State Examination, MMSE; Clinical Dementia Rating, CDR), and pathological status (Consortium to Establish Registry for Alzheimer’s Disease (CERAD) neuritic plaque score, Braak stage) are provided for subjects with frontal cortical and hippocampal slices in Table 1. Compared to CN subjects (median age 93.5 years, intra-quartile range (IR) [89.3, 98.2]) and MCI subjects (median age 94.9 years, IR [93.0, 98.8]), subjects with AD were significantly younger (median age 88.7 years, IR [81.3, 92.3]; *P* = 0.031, *P* = 0.003, respectively; 1-way ANOVA with Tukey’s post hoc correction). These groups did not differ in years of education or gender distribution. They exhibited expected patterns of *APOE4* allele distribution, with more *APOE4* carriers among the AD group. Expected differences were observed in MMSE, CDR, CERAD score, and Braak stage between CN and MCI groups but were not significant. AD groups exhibited expected reductions in cognitive and functional status, and higher CERAD scores and Braak stages.

**Fig. 1:**
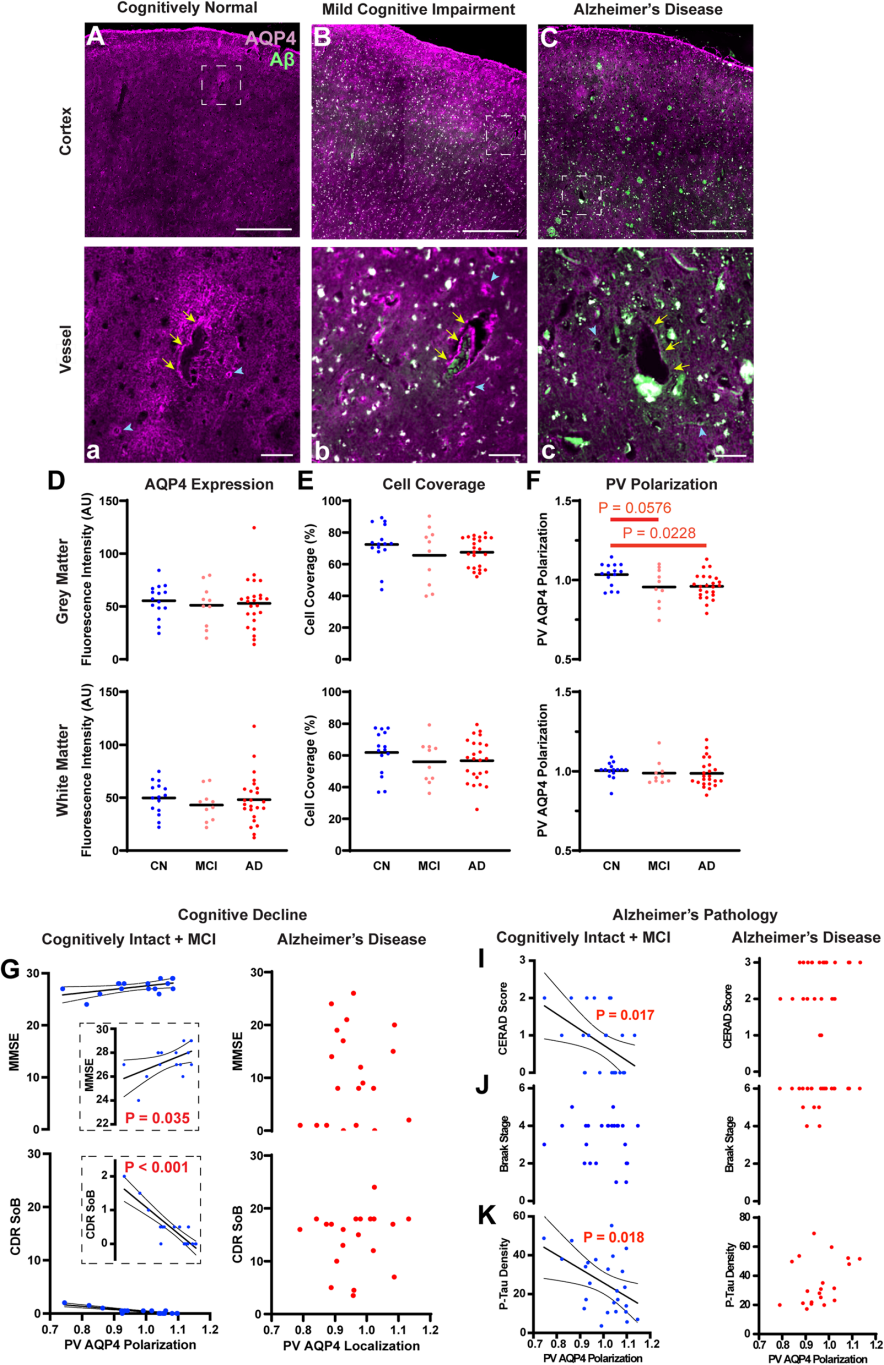
Loss of perivascular AQP4 localization is associated with pathology and cognitive impairment in the early stage Alzheimer’s disease. **A–C**, **a–c** Representative images of the immunofluorescent labeling of AQP4 and Aβ in frontal cortex of cognitively normal (CN, **A**, **a**), mild cognitive impairment (MCI, **B**, **b**), and Alzheimer’s disease (AD, **C, c**) subjects. The bottom panel (**a–c**) shows the areas indicated by white squares in the top panel (**A–C**) at higher magnification. Yellow arrows indicated the large vessels and blue arrow heads indicate the microvessels. Scale bar: 1 mm (**A–C**) and 0.1 mm (**a–c**). In CN subjects, AQP4 expression was largely uniform through the cortical layers between the pial surface and subcortical white matter boundary (**A**), with increased IF at perivascular endfeet surrounding the cerebral vasculature (**a**). In MCI and AD subjects, AQP4 expression became more irregular (**B–C**, **b–c**). Quantification of mean AQP4 IF (**D**) and cell coverage (**E**) showed comparable values between CN, MCI, and AD groups frontal cortical grey matter (top) and white matter (bottom). **F** Perivascular polarization was significantly reduced in AD compared to CN subjects in frontal cortical gray matter (top; *P* = 0.0228, one-way ANOVA with Dunnett’s post hoc test), but not white matter (bottom). Among non-demented (CN + MCI) subjects, reduced perivascular localization was associated with cognitive and functional decline measured by Mini-Mental State Examination (MMSE, *P* = 0.035, R^2^ = 0.279) (**G**) and Clinical Dementia Rating Sum of Boxes (CDR SoB, *P* < 0.001, R^2^ = 0.7832) scores (**H**). No associations were observed in subjects with AD. Among non-demented (CN + MCI) subjects (left), the perivascular AQP4 polarization was significantly associated with the cortical amyloid β plaque density measured by the CERAD neuritic plaque score (**I**) (*P* = 0.017, R^2^=0.224). No association was observed with the anatomical Braak staging of tau pathology (**J**); however, increased frontal cortical P-tau IF was associated with reduced perivascular AQP4 localization (**K**, *P* = 0.018, R^2^ = 0.220). Among AD subjects, no such associations were observed (right). 95% confidence intervals are shown for significant findings

Immunofluorescent double labeling for AQP4 and Aβ (4G8 clone) was conducted in both frontal cortical and hippocampal slices. Representative wide-field fluorescence micrographs show that AQP4 IF in CN subjects was largely uniform through the cortical layers between the pial surface and subcortical white matter boundary, with increased IF at perivascular endfeet surrounding the cerebral vasculature (Fig. 1A). In MCI and AD subjects, cortical AQP4 IF appeared more “patchy” and varied between cortical layers (Fig. 1B,C). Quantification of mean AQP4 IF revealed no consistent differences in relative AQP4 expression in the frontal cortical gray matter or white matter between CN, MCI, or AD subjects (Fig. 1D), and no differences in the CA1, CA2, or CA3 hippocampal subfields between CN, MCI, and AD subjects (Supplemental Figure 3A). As described in Supplemental Figure 1, the observed “patchiness” was evaluated by thresholding analysis to calculate the area coverage of cellular AQP4 IF. Using this approach, although cellular AQP4 IF coverage tended to be lower in MCI and AD subjects, measured differences between CN, MCI, and AD subjects were not statistically significant. No significant differences were observed between CN, MCI, and AD subjects in either frontal cortex (Fig. 1E), or in the CA2 or CA3 hippocampal subfields (Supplemental Figure 4B). AQP4 cell coverage was reduced in the CA1 subfield of MCI compared to CN subjects (Supplemental Figure 4B, *P* = 0.0086, one-way ANOVA with Dunnett’s post hoc correction).

Perivascular AQP4 polarization was quantified as the ratio between AQP4 IF along perivascular endfeet and the intensity in the cellular compartment (as detailed in Supplemental Figure 1). Perivascular AQP4 polarization in the frontal cortical gray matter was significantly reduced in AD compared to CN subjects (Fig. 1F; *P* = 0.0228, one-way ANOVA with Dunnett’s post hoc test). A similar reduction was observed in MCI compared to CN subjects, but this difference was not significant with correction for multiple comparisons (*P* = 0.0576, one-way ANOVA with Dunnett’s post hoc test). No significant differences in perivascular AQP4 polarization were observed in the frontal cortical white matter (Fig. 1F) or in the CA1, CA2, or CA3 hippocampal subfields (Supplemental Figure 4C).

We evaluated the association between changes in perivascular AQP4 polarization in the frontal cortical gray matter and measures of global cognitive function. When all subjects were evaluated without regard to clinical diagnosis, no significant association was observed between AQP4 localization and MMSE (*P* = 0.2852) or the CDR Sum of Boxes (CDR SoB) score (*P* = 0.455). Because *Aqp4* deletion promotes Aβ deposition [18], proposed to be an early event in the Alzheimer’s pathological cascade [31], we evaluated whether changes in perivascular AQP4 localization were associated with cognitive decline prior to the development of frank dementia. Among non-demented subjects (CN + MCI), lower perivascular AQP4 localization was associated with lower MMSE scores (*P* = 0.035, R^2^ = 0.279) and higher CDR SoB scores (*P* < 0.001, R^2^ = 0.7832, Fig. 1G–H). Among subjects with AD, no association with MMSE (*P* = 0.720) or CDR SoB (*P* = 0.704) was observed.

We next evaluated the association between perivascular AQP4 polarization in the frontal cortical gray matter and either global or local measures of Alzheimer’s-related Aβ and p-tau pathology. Among non-demented (CN + MCI) subjects, reduced perivascular AQP4 localization was associated with higher CERAD neuritic plaque score, an ordinal measure of cortical Aβ plaque density (Fig. 1I, *P* = 0.017, R^2^=0.224). No association was observed among subjects with AD diagnosis. No association was observed between changes in perivascular AQP4 localization and Braak stage in either non-demented or subjects with AD (Fig. 1J, *P* = 0.328, *P* = 0.388, respectively). Because Braak staging assesses topographic spread rather than local intensity or density of p-tau pathology, we further evaluated pathological phosphorylated tau (p-tau) levels by IF using the AT8 (Ser202/Thr205, human/mouse cross-reactive) antibody in the frontal cortex and hippocampus. As expected, p-tau density was significantly increased in the frontal cortical gray matter (Supplemental Figure 5D, *P* = 0.011, 2-way ANOVA with Tukey’s post hoc test) and CA1 and CA2 subfields (*P* < 0.001) of AD subjects (Supplemental Figure 5A-D). Similarly, frontal cortical p-tau density was positively correlated with Braak stage (Supplemental Figure 5E, *P* = 0.011, R^2^ = 0.139). Among non-demented (CN+MCI) subjects, reduced frontal cortical perivascular AQP4 polarization was associated with increased frontal cortical p-tau IF (Fig. 1K, *P* = 0.018, R^2^ = 0.220), but no association was observed among subjects with AD (*P* = 0.172). These data demonstrate that subjects with AD exhibit reduced perivascular AQP4 polarization in the frontal cortical gray matter and that prior to the onset of dementia, loss of perivascular AQP4 polarization is associated with cognitive decline and local frontal cortical Aβ and tau pathology.

We next performed linear regression analysis to control for cognitive status in CN and MCI individuals (cognitive status as a covariate). Using this approach, reduced frontal cortical perivascular AQP4 localization was not significantly associated with reduced MMSE score (*P* = 0.200, adjusted (Adj) R^2^ = 0.201), but was associated with higher CDR SoB score (*P* < 0.0001, Adj R^2^ = 0.805) (Supplemental Figure 6A-B). Reduced perivascular AQP4 localization was associated with CERAD neuritic plaque score (*P* = 0.024, Adj R^2^ = 0.156), was not significantly associated with Braak stage (*P* = 0.613, Adj R^2^ = 0.002), and was significantly associated with local p-tau density (*P* = 0.030, Adj R^2^ = 0.149) (Supplemental Figure 6C-E).

### Age-related changes in perivascular AQP4 localization in the mouse brain

**Fig. 2:**
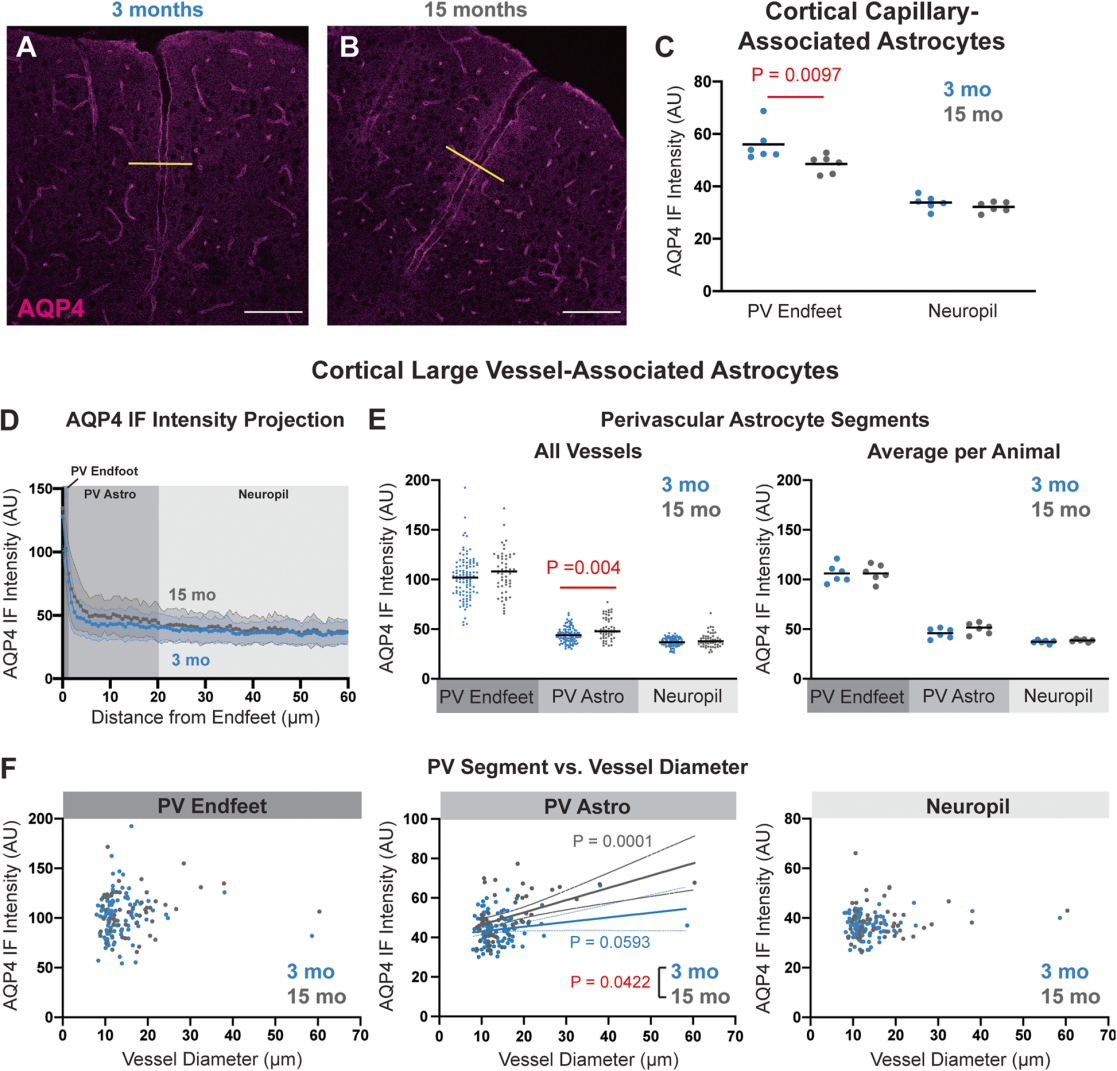
Age-related changes in the perivascular AQP4 polarization in the mouse cortex (**A,B**): Representative confocal micrographs of AQP4 IF labeling in 3-month-old (**A**) and 15-month-old (**B**) mouse cortex. Scale bars: 100 μm. **C** In capillary-associated astrocytes, the AQP4 IF at the perivascular (PV) endfeet was significantly reduced in aged animals (*P* = 0.0097, mixed effects model with Sidak post hoc test), while the AQP4 IF in the surrounding non-perivascular neuropil was unchanged. **D** Cross-sectional AQP4 IF projections (yellow lines in **A**,**B**) across large cortical vessels were quantified and averaged between 3-month (blue, 104 vessels from 6 animals) and 15-month (grey, 55 vessels from 6 animals) old mice. **E** Segmentation of projections into PV Endfeet (0–1.5 μm from the vessel wall), the PV Astrocyte (1.5–20 μm from the vessel wall), and the surrounding neuropil (20–68 μm from the vessel wall) showed that along large cortical vessels, PV Astrocyte AQP4 IF was significantly higher in the aged mice (*P* = 0.004, mixed effects model with Sidak post hoc correction). Plot at left shows values for all vessels measured, plot at right shows averaged values per animal. **F** Among large cortical vessels, PV Endfoot (left) or Neuropil (right) IF was not related to vessel diameter. PV Astrocyte IF was significantly associated with increasing vessel diameter (middle). This association was significantly (*P* = 0.0422) steeper for 15-month-old (gray; *P* = 0.0001, R^2^ = 0.2410) compared to 3-month-old animals (blue; *P* = 0.0593, R^2^ = 0.03444)

We next sought to replicate and extend findings from our prior study [13] to define whether similar changes in AQP4 localization are observed in the aging rodent brain as are observed in aging and AD human subjects ([20], Fig. 1). IF and confocal imaging revealed that as previously reported, cortical AQP4 in 3-month-old animals is predominantly perivascular (Fig. 2A) while in 15-month-old animals, there is an increase in diffuse AQP4 IF in astrocytes surrounding the penetrating vasculature (Fig. 2B). Using fluorescent lectin staining to label the cerebrovascular endothelium, we measured AQP4 IF in capillary-associated perivascular endfeet (“PV Endfoot”) and AQP4 IF in the surrounding non-perivascular neuropil (Supplemental Figure 2). Evaluating AQP4 IF at the level of capillaries, we found that microvascular perivascular AQP4 IF was significantly reduced in 15 compared to 3-month-old mice, while the non-perivascular AQP4 IF was not impacted by age (Fig. 2C, *P* = 0.0097, mixed effect model with Sidak post hoc test). We evaluated the AQP4 IF profile surrounding penetrating and large intracortical vessels (both arteries and veins). Segmenting the signal into three compartments: the immediate PV Endfoot region (0–1.5 μm from the vessel wall, “PV Endfoot”), the layer of astrocytes immediately bounding these penetrating vessels (“PV Astro,” 1.5–20 μm from the vessel wall), and the neuropil remote from these vessels (20–68 μm from the vessel wall), we observed that while PV Endfeet surrounding these large vessels did not differ in AQP4 IF between 3- and 15-month-old animals, the layer of astrocytes immediately surrounding these penetrating vessels exhibited increased diffuse AQP4 IF throughout their fine non-perivascular processes in 15 compared to 3-month-old animals (Fig. 2E, *P* = 0.004, mixed effect model with Sidak post hoc correction). When we evaluated the relationship between vessel diameter and AQP4 IF in these three segments, we observed that diffuse AQP4 IF in the PV Astro segment was significantly associated with increasing vessel diameter and that the correlation is more positive in 15-month-old mice than in 3-month-old mice (*P* = 0.0422; R^2^_15mo_ = 0.2410, R^2^_3mo_ = 0.03444) (Fig. 2F).

We used the same approach to evaluate the effect of age on AQP4 localization in the hippocampal microcirculation and large vessels of the stratum lacunosum moleculare (Supplemental Figure 7A-B). Unlike the cortex, there was no effect of age on capillary-associated perivascular AQP4 IF nor on non-perivascular AQP4 IF (Supplemental Figure 7C). Cross-sectional analysis of large vessels showed no change in AQP4 IF in PV Endfeet with age, but as in the cortex, increased AQP4 IF in astrocytes surrounding large vessels (PV Astro) was detected in 15-month-old compared to 3-month-old animals (Supplemental Figure 7D-E, *P* = 0.0003, mixed effect model with Sidak post hoc test). No significant association was observed between AQP4 IF in any segment and vessel diameter at either age (Supplemental Figure 7F). Together, these findings demonstrate that as in the aging and AD human cortex, two distinct changes in AQP4 localization occur in the aging rodent cortex: first, a loss of perivascular localization surrounding the cortical microcirculation, and second, an enhancement of AQP4 distribution over the fine, non-perivascular processes of astrocytes surrounding large penetrating vessels in both the cortex and hippocampus.

### Viral AQP4-M1 overexpression increases fine process AQP4 localization in mice

We next sought to model the distribution of AQP4 to the fine processes in astrocytes surrounding large penetrating vessels in the cortex and hippocampus, one of the phenotypic changes observed in the aging mouse (Fig. 2) as well as the aged and AD human cortex ([20], Fig. 1), using AAV-based gene delivery. All AAV constructs used in the present study are detailed in Supplemental Table 1. The two most abundant AQP4 isoforms, AQP4-M1 and AQP4-M23, exhibit divergent membrane localization profiles when overexpressed on an *Aqp4-*null background in primary astrocytes, with AQP4-M1 dispersing over the cell surface and AQP4-M23 forming large clusters within the cell membrane [32]. We first sought to confirm whether this was also observed following overexpression on a wild type *Aqp4* background both in vitro and in vivo. We utilized an AAV with an astrocyte-specific promoter (AAV8-GfaABC1D) to drive expression of c-terminus enhanced green fluorescent protein (eGFP)-tagged versions of AQP4-M1 (AQP4-M1-eGFP; AAV8-M1-eGFP) and AQP4-M23 (AQP4-M23-eGFP; AAV8-M23-eGFP) in cultured primary mouse cortical astrocytes (Supplemental Figure 8A-B). Consistent with prior studies, overexpression of AQP4-M1-eGFP resulted in punctate green fluorescence distributed evenly over the cell surface, while overexpression of AQP4-M23-eGFP resulted in larger fluorescence puncta than those for AQP4-M1-eGFP (Supplemental Figure 8C-D). Puncta sizes were quantified, revealing that AQP4-M23-eGFP overexpression increased the ratio of large puncta (≥2 μm^2^) to small puncta (≤1 μm^2^) compared to that observed in AQP4-M1-eGFP overexpressing cells (Supplemental Figure 8E, *P* = 0.0540 unpaired *t-*test).

We next evaluated whether these differences in AQP4 membrane cluster size related to perivascular AQP4 localization in vivo. Using local intracortical injection of AAV8-M1-eGFP or AAV8-M23-eGFP, with in vivo 2-photon microscopy, perivascular localization of eGFP-tagged AQP4 was evaluated in reference to the cerebral vasculature labeled with an intravascular fluorescent tracer (70 kD Texas Red-conjugated dextran). AAV8-M1-eGFP or AAV8-M23-eGFP injection resulted in widespread cortical astroglial AQP4-eGFP expression (Supplemental Figure 8F). Visualization of individual astrocytes showed that AQP4-M1-eGFP overexpression resulted in small fluorescence puncta dispersed throughout astroglial fine processes, while AQP4-M23-eGFP puncta were larger in size (Supplemental Figure 8G). When perivascular compared to fine process localization of GFP fluorescence was evaluated, we observed that AQP4-M23-eGFP was significantly more perivascular in its localization than AQP4-M1-eGFP (Supplemental Figure 8H, *P* = 0.0457, unpaired *t*-test).

**Fig. 3:**
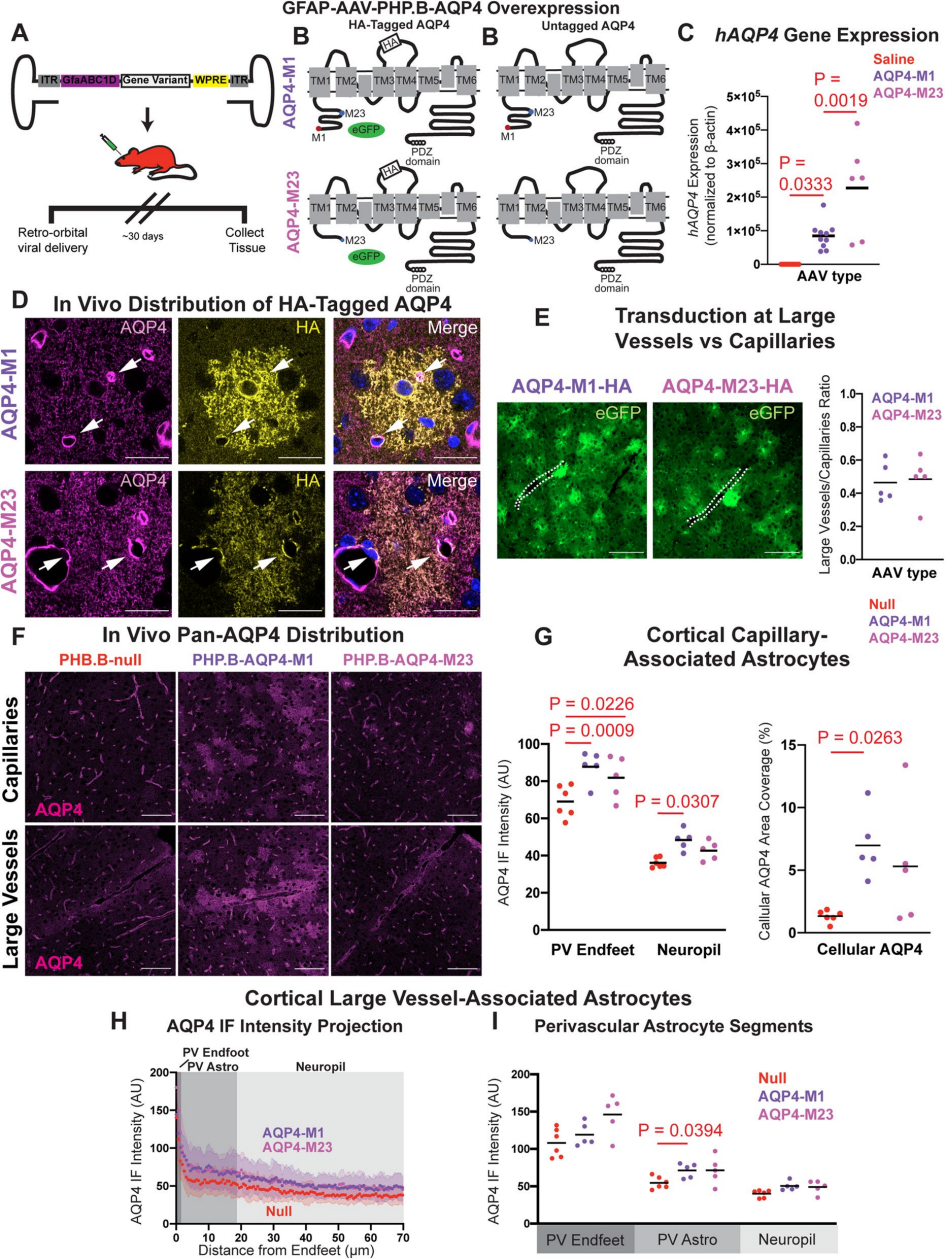
Viral AQP4-M1 overexpression increases cellular AQP4 localization in mouse cortex. **A** Schematic outline of the viral approach to overexpress AQP4-M1 or -M23 isoforms under the astrocyte-specific GfaABC1D promoter in vivo. **B** AAV^PHP^ constructs were generated to drive expression of untagged human AQP4 (hAQP4)-M1 and -M23 isoforms (right), or co-expression of HA-tagged hAQP4-M1 and -M23 with enhanced green fluorescent protein (eGFP) reporter. **C** Thirty days after retro-orbital viral delivery, qPCR showed that untagged hAQP4-M1 and hAQP4-M23 were robustly expressed in the mouse cortex (*P* = 0.0333, *P* = 0.0019; one-way ANOVA with Tukey’s post hoc correction). **D** Thirty days after iv viral delivery, HA-tagged AQP4-M1 exhibited no specific perivascular (white arrows) localization, distributing over all fine processes (top) while HA-tagged AQP4-M23 localized to both perivascular endfeet and fine astroglial processes (bottom). Scale bar: 20 μm. **E** Evaluation of eGFP reporter expression showed that viruses driving AQP4-M1 (top image) and AQP4-M23 (bottom image) efficiently transduced astrocytes surrounding both large cortical vessels (white dash lines) and capillaries, and that the transduction ratios along large vessels and capillaries were comparable (panel at right). Scale bar: 100 μm. **F** Thirty days after delivery of virus driving untagged AQP4-M1 and -M23, AQP4 IF was evaluated with a pan-AQP4 antibody in capillaries (top) and large cortical vessels (bottom). AQP4-M1 overexpression resulted in a diffuse pattern of AQP4 labeling in the fine processes of astrocytes throughout the cortex while AQP4-M23 overexpression did not result in marked cellular labeling. Scale bar: 100 μm. **G** In cortical capillary-associated astrocytes, overexpression of AQP4-M1 isoform significantly increased the AQP4 IF intensity at both PV Endfeet and in the surrounding non-perivascular neuropil (Left, *P* = 0.0009, *P* = 0.0307, respectively. 2-way ANOVA with Tukey’s post hoc test), as well as the cellular AQP4 area coverage (Right, *P* = 0.0263, 1-way ANOVA with Tukey’s post hoc test). On the contrary, overexpression of AQP4-M23 isoform only increased the AQP4 IF intensity at the PV Endfeet (left; *P* = 0.0226, 2-way ANOVA with Tukey’s post hoc test). **H**,**I** Cross-sectional analysis of AQP4 IF surrounding large cortical vessels showed that compared to control (null) virus (30 vessels from 6 animals), AQP4-M1 (31 vessels from 5 animals) overexpression significantly increased AQP4 IF in PV Astrocytes segments (*P* = 0.0394, 2-way ANOVA with Tukey’s post hoc test) while AQP4-M23 (27 vessels from 5 animals) did not

To assess the localization of AQP4 isoforms in vivo without an eGFP tag, we utilized a second AAV variant that efficiently crosses the blood-brain barrier and exhibits a high trophism for astrocytes, coupled with an astrocyte-specific promoter, AAV-PHP.B-GfaABC1D [33]. We generated one AAV^PHP^ eGFP reporter and four AAV^PHP^ AQP4 viral constructs. The reporter construct drove expression of eGFP under the astrocyte-specific GfaABC1D promoter (AAV^PHP^-eGFP). The next two constructs drove expression of unlabeled AQP4-M1 (AAV^PHP^-M1) or AQP4-M23 (AAV^PHP^-M23) under control of this promoter. To distinguish between background and overexpressed AQP4 expression, the final two constructs used P2A sites to co-express AQP4 isoforms tagged with a hemagglutinin (HA) tag on the second external loop of the AQP4 protein, as well as an eGFP reporter (AAV^PHP^-M1-HA, AAV^PHP^-M23-HA) (Fig. 3A,B).

Thirty days after intravenous injection of the eGFP reporter construct (1 × 10^12^ vg), eGFP expression was observed throughout the brain, including in all layers of the cerebral cortex and in subcortical structures (Supplemental Figure 9A). Immunofluorescent triple-labeling with the astroglial marker GFAP and the neuronal marker NeuN showed that eGFP expression was restricted to stellate GFAP-positive astrocytes and was not expressed in NeuN-positive neurons (Supplemental Figure 9B). Immunofluorescent labeling also demonstrated that GFP reporter expression was not present in CD31-positive endothelial cells (Supplemental Figure 9C-D).

Thirty days following retro-orbital virus injection of AAV^PHP^-M1 or AAV^PHP^-M23, robust expression of human AQP4 message was observed by qPCR (Fig. 3C). We next used AAV^PHP^-M1-HA and AAV^PHP^-M23-HA constructs to evaluate the localization of AQP4 isoforms after systemic virus administration. We observed widespread cortical and hippocampal expression of HA-tagged AQP4 with both constructs 30 days after injection. Super-resolution microscopy targeting individual eGFP-expressing cortical astrocytes following staining with a pan-AQP4 antibody (recognizing both wild type mouse and overexpressed human AQP4) revealed that overall, AQP4 localized preferentially to perivascular astrocytic endfeet. However, detection of the HA-human isoform using an anti-HA antibody showed no specific perivascular localization, but rather AQP4 distributed over all fine processes (Fig. 3D, top). In contrast, HA-tagged AQP4-M23 did localize to both perivascular endfeet and fine astroglial processes (Fig. 3D, bottom). Enhanced GFP expression was evaluated for enrichment proximal to large vessels or within capillary beds. Transduction of both large vessel-adjacent and capillary-adjacent astrocytes was observed for both M1 and M23 viruses (Fig. 3E, left). Quantification of the ratio of capillary-adjacent astrocytes to large vessel-adjacent astrocytes revealed a 2.24-fold higher transduction of capillary astrocytes compared to large vessel that was similar between both viruses (Fig. 3E, right). Evaluation of eGFP also permitted assessment of the extent and specificity of construct expression. As observed with the AAV^PHP^-eGFP reporter (Supplemental Figure 9), eGFP distributed widely in volume-filling cells with morphology consistent with astrocyte-specific expression (Fig. 3D,E).

We next measured the effect of untagged AQP4-M1 and AQP4-M23 overexpression on overall perivascular localization of cortical and hippocampal AQP4. Immunofluorescence with a pan-AQP4 antibody followed by confocal imaging revealed that AQP4-M1 overexpression resulted in a diffuse pattern of AQP4 labeling in the fine processes of astrocytes throughout the cortex (Fig. 3F) and hippocampus (Supplemental Figure 10A). Quantification of AQP4 localization around cortical capillaries revealed that compared to injection of null vector, both AQP4-M1 and AQP4-M23 overexpression increased AQP4 expression at PV Endfeet (Fig. 3G, left; *P* = 0.0009, *P* = 0.0226, respectively, 2-way ANOVA with Tukey’s post hoc test). The non-perivascular neuropil AQP4 IF intensity and the cellular AQP4 coverage were significantly increased in the cortex of AQP4-M1-overexpressng animals (Fig. 3G, left; *P* = 0.0307, 2-way ANOVA with Tukey’s post hoc test, right; *P* = 0.0263, 1-way ANOVA with Tukey’s post hoc test) and similar results were observed in the hippocampus (Supplemental Figure 10B). When AQP4 localization surrounding large penetrating vessels was evaluated, we observed that while AQP4-M1 and AQP4-M23 overexpression did not alter AQP4 IF at PV Endfeet or in the surrounding neuropil, significantly increased AQP4 expression in the astrocytes surrounding large vessels (PV Astro) was observed following AQP4-M1 overexpression (Fig. 3H,I, *P* = 0.0394, 2-way ANOVA with Tukey’s post hoc test). AQP4-M1 overexpression did not alter AQP4 localization in astrocytes surrounding the large vessels of the hippocampal stratum lacunosum moleculare (Supplemental Figure 10C-D). In total, these data demonstrate that AAV-based overexpression of AQP4-M1 is a suitable approach for modeling the increased fine process AQP4 expression observed surrounding large vessels in the aging rodent brain (Fig. 2) [13].

### Increased fine process AQP4 does not impair CSF-ISF exchange or alter amyloid β levels in the mouse brain

**Fig. 4:**
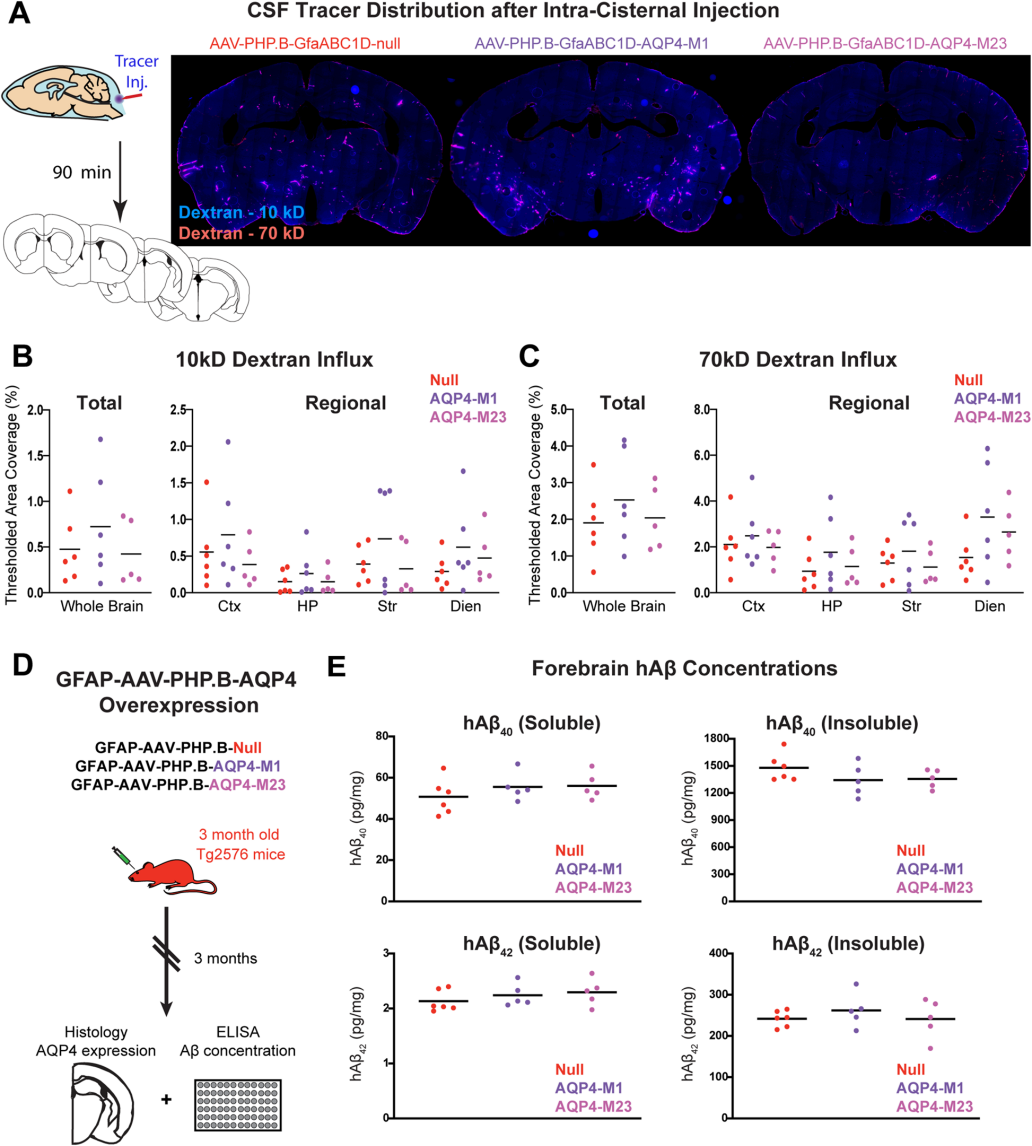
Increased fine process AQP4 does not impair perivascular CSF-ISF exchange or alter amyloid β deposition. **A** Schematic outline of the workflow of the intracisternal tracer injection followed by the tracer visualization (left). Representative images of 70-kD CSF tracer distribution 90 min following injection are shown in animals 28 days after virus injection at right. Quantification of CSF tracer distribution showed that neither AQP4-M1 nor AQP4-M23 overexpression significantly altered 10 kD (**B**) or 70 kD (**C**) CSF tracer influx either into the whole brain, or into four subregions (cortex, hippocampus, striatum, and the diencephalon) compared to those injected with control virus. **D** Schematic outline of the workflow to define the effect of AQP4-M1 and AQP4-M23 overexpression on Aβ accumulation. Tg2576 mice at 3 months of age were injected with AAV^PHP^-M1, AAV^PHP^-M23, or null vector. Three months later, whole-brain human Aβ levels were assessed by ELISA. **E** At 6 months of age, no differences were observed in either hAβ_1-40_ or hAβ_1-42_ levels in either soluble or insoluble fractions from animals overexpressing AQP4-M1 or AQP4-M23 compared to null vector-injected controls

Next, we sought to measure whether increasing fine process AQP4 localization by AQP4-M1 overexpression is sufficient to recapitulate the impairment in CSF tracer influx observed previously in aging mice [13]. Twenty-eight days after AAV^PHP^-M1, AAV^PHP^-M23, or null vector injection, 10 kD (Cascade Blue conjugate) and 70 kD (Texas Red conjugate) dextrans were co-infused intracisternally into the CSF of wild type animals. Ninety minutes later, the tissue was perfusion-fixed and parenchymal tracer influx was evaluated by imaging five coronal sections across the cerebrum (1, 0, − 1.5, − 2.5, and − 3.5 mm relative to bregma). Overexpression of either AQP4-M1 or AQP4-M23 had no effect on tracer influx compared to mice treated with null virus for either the 10 kD or 70 kD dextrans (Fig. 4B,C). This was observed both globally, and upon assessment of tracer influx into individual subregions (cortex, hippocampus, striatum, and the diencephalon).

To determine whether increased AQP4 localization to diffuse fine processes alters Aβ levels, we determined whether expression of AQP4 using an AAV-based approach would alter Aβ burden in Tg2576 mice [23]. Three-month-old Tg2576 mice were injected with AAV^PHP^-M1, AAV^PHP^-M23, or null vector, and 3 months later, Aβ levels were evaluated in the parenchyma (Fig. 4D). Assessment of AQP4 localization at this time point in AAVPHP-M1-treated Tg2576 mice showed that AQP4-M1 overexpression and the distribution of AQP4 to astroglial fine processes persisted at this time point (Supplemental Figure 11A-B). No differences were observed in either Aβ_1-40_ or Aβ_1-42_ levels with AQP4-M1 or AQP4-M23 overexpression compared to null vector-injected controls (Fig. 4E). These data suggest that increasing fine process AQP4 expression with viral AQP4-M1 overexpression does not appreciably alter either perivascular exchange or Aβ levels in the Tg2576 model.

### Loss of perivascular AQP4 localization impairs CSF-ISF exchange and increases Aβ levels in the mouse brain

**Fig. 5:**
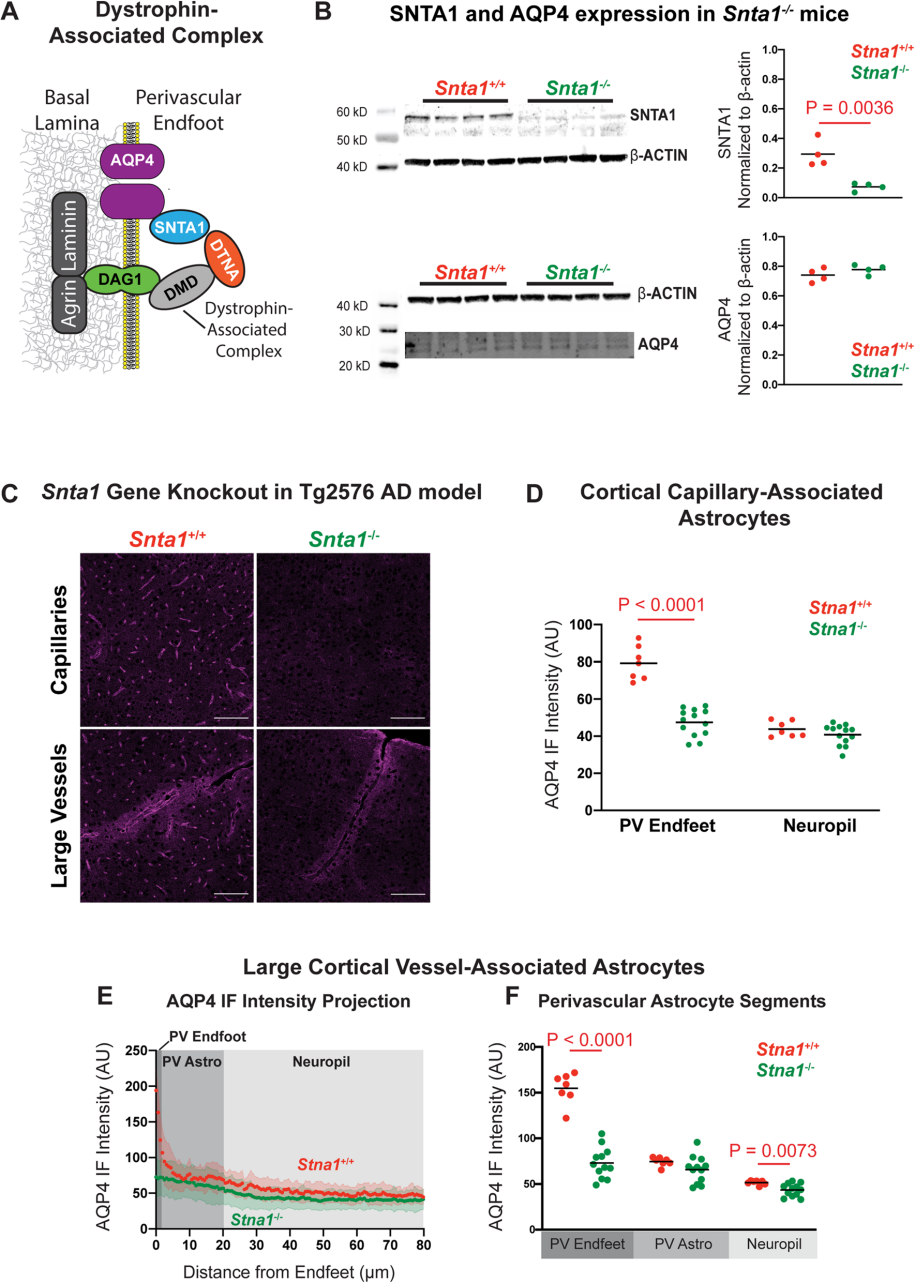
*Snta1* gene deletion eliminates perivascular AQP4 localization. **A** Schematic outline of dystrophin-associated complex that includes α-syntrophin (SNTA1), dystrobrevin (DTNA), dystrophin (DMD), and dystroglycan (DAG1) and anchors AQP4 to the perivascular astrocyte endfeet. **B** Western blot demonstrates that *Snta1* gene deletion abolishes SNTA1 protein expression (top; *P* = 0.0036, unpaired *t*-test), while total AQP4 expression remained unchanged (bottom). **C** Representative images of the cortical AQP4 expression along capillaries (top) and large vessels (bottom) in the wild type and *Snta1*^−/−^ mouse cortex shows complete loss of perivascular AQP4 localization with *Snta1* deletion. Scale bar: 100 μm. **D** In cortical capillary-associated astrocytes, AQP4 IF in PV Endfeet is reduced to levels of the non-perivascular neuropil in *Snta1*^−/−^ mice (*P* < 0.0001, mixed effects model with Sidak’s post hoc test), while the neuropil AQP4 IF remained comparable between *Snta1*^−/−^ and *Snta1*^+/+^ mice. **E**,**F** The cross-sectional AQP4 IF along large cortical vessels of showed reduced PV Endfoot AQP4 IF (*P* < 0.0001, respectively, mixed effects model with Sidak’s post hoc test) in *Snta1*^−/−^ mice (94 vessels from 12 animals) compared to *Snta1*^+/+^ mice (44 vessels from 7 animals). Non-perivascular neuropil AQP4 IF was also significantly reduced in *Snta1*^−/−^ compared to *Snta1*^+/+^ mice (*P* = 0.0073)

We next sought to model the loss of microvascular perivascular AQP4 localization observed in the cortex of human aged and AD subjects ([20], Fig. 1), and aging mice (Fig. 2). The localization of AQP4 to perivascular astroglial endfeet results from its binding to the dystrophin-associated complex, a multi-protein complex including α-syntrophin (SNTA1), dystrobrevin, dystrophin, and dystroglycan linking AQP4 to the extracellular matrix of the basal lamina (Fig. 5A). Prior studies demonstrate that deletion of the α-syntrophin gene (*Snta1*) eliminates the perivascular localization of AQP4 [25, 34]. In our hands, Western blot demonstrated that *Snta1* deletion abolishes SNTA1 expression (Fig. 5B, *P* = 0.0036, *t*-test). As reported in prior studies [35, 36], total AQP4 expression was not significantly different between *Snta1*^+/+^ and *Snta1*^−/−^ mice (Fig. 5B). Immunofluorescence and confocal microscopy revealed a virtually complete loss of perivascular AQP4 localization surrounding the cerebral microcirculation (Fig. 5C), which was reflected in a dramatic reduction in capillary-associated PV Endfoot AQP4 IF (Fig. 5D, *P* < 0.0001, mixed effect model with Sidak’s post hoc test) with no change in AQP4 IF in the surrounding non-perivascular neuropil. Evaluation of AQP4 localization associated with large vessels showed that *Snta1* gene deletion eliminated AQP4 enhancement at the PV endfeet (Fig. 5E,F; Supplementary Figure 10B-C) but did not alter the increased expression of AQP4 in astrocytes surrounding large penetrating vessels. Within the hippocampus, *Snta1* gene deletion abolished perivascular AQP4 localization associated with the microvasculature (Supplemental Fig. 12A,B) but did not alter AQP4 localization at the endfeet surrounding the large vessels of the stratum lacunosum moleculare (Supplemental Figure 12C-D). This latter finding suggest that localization of AQP4 to the perivascular endfeet surrounding this population of vessels may be dependent upon a different set of adaptor proteins than around other vessels. More generally, these findings demonstrate that the *Snta1*-null mouse models well the loss of perivascular endfoot AQP4 localization, but not the increase in non-perivascular fine process AQP4 expression, observed in the aging brain.

**Fig. 6:**
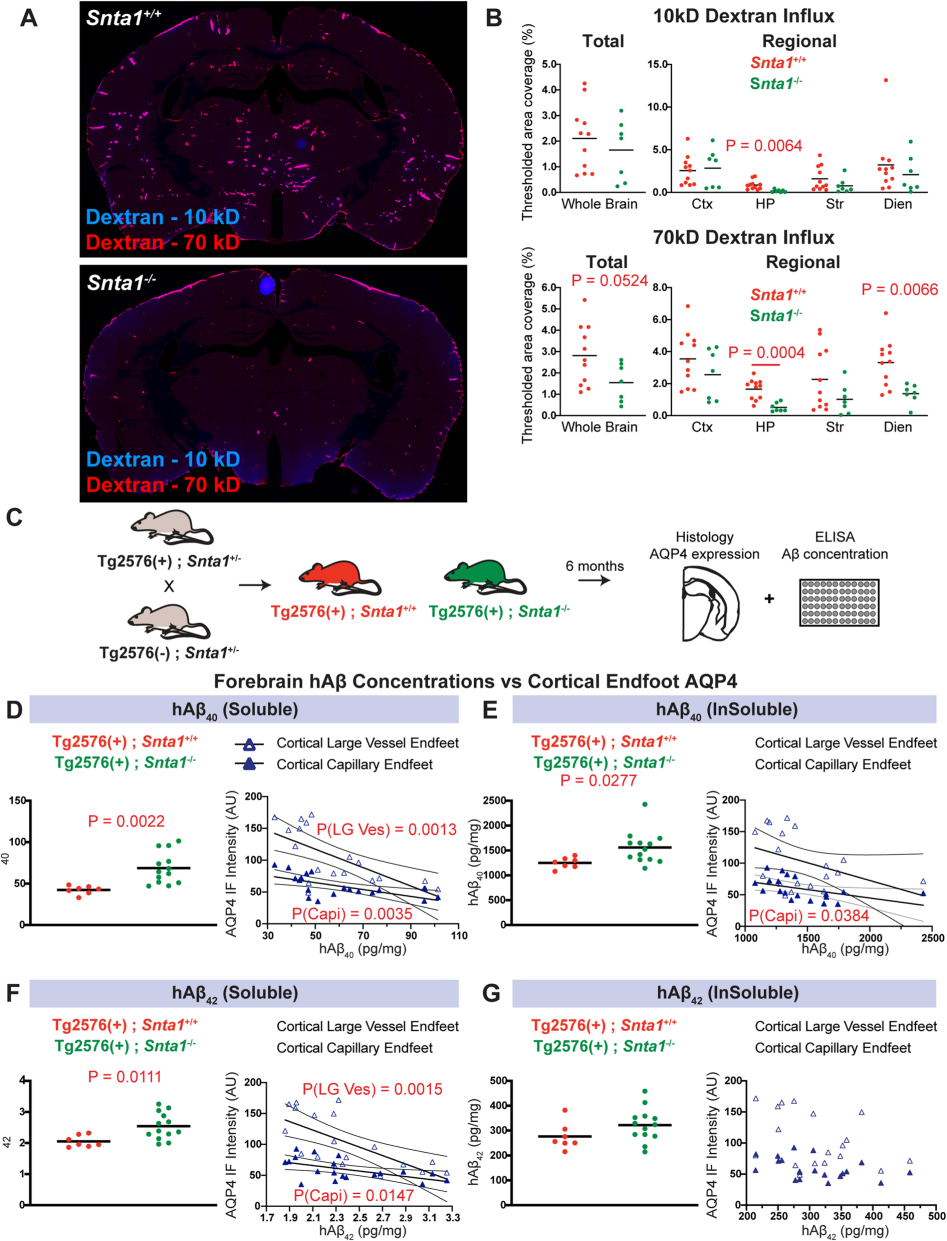
Loss of perivascular AQP4 localization impairs CSF-ISF exchange and promotes amyloid β deposition. **A** Representative images of the 10 kD (top) and 70 kD (bottom) CSF tracer distribution in the wild type and *Snta1*^−/−^ mice 90 min after intracisternal injection of 10 kD (Cascade Blue) or 70 kD (Texas Red) dextrans. **B** CSF tracer area coverage was generally reduced in *Snta1*^−/−^ compared to wild type mice (*P* = 0.0524, unpaired *t*-test), with greatest reductions in hippocampus (*P* = 0.0062, 2-way ANOVA with Sidak’s post hoc test). Influx of 70 kD tracer was reduced to a greater extent, with greatest declines in hippocampus and diencephalon (*P* = 0.0004, *P* = 0.0065, respectively, 2-way ANOVA with Sidak’s post hoc test). **C** Schematic outline of study evaluating impact of AQP4 localization on Aβ deposition in the Tg2576 line. At 6 months of age, soluble Aβ_40_ (**D**; *P* = 0.0022, unpaired *t*-test) and insoluble Aβ_40_ (**E**; *P* = 0.0277, unpaired *t*-test) were significantly increased in Tg2576 (+); *SNTA1*^−/−^ compared to Tg2576 (+); *SNTA1*^+/+^ littermates. Soluble Aβ_42_ (**F**; *P* = 0.0022, unpaired *t*-test) levels were increased in Tg2576 (+); *SNTA1*^−/−^ compared to Tg2576 (+); *SNTA1*^+/+^ littermates, although no significant change in insoluble Aβ_42_ was observed (**G**). **D–G** Regression analysis showed that reduced capillary-associated PV Endfoot AQP4 IF was associated with increasing soluble Aβ_40_ (*P*_Capi_ = 0.0035, R^2^_Capi_ = 0.4030), insoluble Aβ_40_ (*P*_Capi_ = 0.0384, R^2^_Capi_ = 0.2286), and soluble Aβ_42_ (*P*_Capi_ = 0.0147, R^2^_Capi_ = 0.3024)**.** Reduced large vessel-associated PV Endfoot AQP4 IF was significantly associated with increasing soluble Aβ_40_ (*P*_LG-Ves_ = 0.0013, R^2^_LG-Ves_ = 0.4651) and soluble Aβ_42_ (*P*_LG-Ves_ = 0.0015, R^2^_LG-Ves_ = 0.4574)

CSF tracer influx into brain tissue was next evaluated using whole-slice fluorescence microscopy following intracisternal tracer injection in both wild type and *Snta1*^−/−^ mice. At 90 min post-injection, penetration of the 10 kD Cascade Blue-conjugated dextran was generally reduced into deep brain structures of the *Snta1*^−/−^ compared to wild type mice. Regional analysis showed that this effect was significant in the hippocampus (Fig. 6A,B, top; *P* = 0.0064, 2-way ANOVA with Sidak’s post hoc test). A trend towards reduced tracer distribution was also observed in the striatum and diencephalon though the difference did not persist following correction for multiple comparisons. Penetration of 70 kD Texas Red-conjugated dextran was impaired in *Snta1*^−/−^ compared to wild type mice, with significant reductions in the hippocampus and diencephalon (Fig. 6B, bottom; *P* = 0.0004, 0.0066, 2-way ANOVA with Sidak’s post hoc test). Consistent with our previously reported dynamic contrast-enhanced MRI studies [17], *Snta1* gene deletion did not significantly reduce CSF tracer distribution when evaluated at 30 min post-injection (data not shown), suggesting that the phenotype of the *Snta1*^−/−^ is relatively muted compared to that observed with global *Aqp4* gene deletion [11, 14, 17, 37].

To define the effect that loss of perivascular AQP4 localization has on interstitial solute efflux, we evaluated the distribution of 10 kD Cascade Blue-conjugated dextran following intraparenchymal injection in *Snta1*^−*/*−^ and wild type mice. Tracer was injected directly into motor cortex and allowed to distribute for 120 min, and interstitial tracer distribution across brain regions was evaluated in fixed coronal slices by whole-slice fluorescence microscopy. Distribution was defined by area coverage of tracer fluorescence within the ipsilateral and contralateral cortex, hippocampus, striatum, and diencephalon integrated through five standard coronal slices per animal (2.5, 1.5, 0, −1, and −2 mm relative to bregma, Supplemental Figure 13A). Following intracortical injection, the 10-kD tracer distributed through the cortex and into the subcortical regions (Supplemental Figure 13B). When compared between wild type and *Snta1*^−/−^ mice at 120 min after injection, tracer fluorescence integrated globally across all slices was generally higher in *Snta1*^−/−^ than in wild type animals (Supplemental Figure 13C-D, *P* = 0.0468, *t*-test), with the most pronounced differences within deeper brain structures such as the striatum (*P* = 0.0340, 2-way ANOVA with Sidak’s post hoc test). These data demonstrate both CSF tracer influx and interstitial tracer efflux are impaired in *Snta1*^−/−^ mice.

To evaluate whether loss of perivascular AQP4 localization increases Aβ levels, we evaluated the effect of *Snta1* gene deletion within the Tg2576 amyloidosis line, comparing soluble and insoluble Aβ levels by ELISA in Tg2576:*Snta1*^−/−^ mice and Tg2576:*Snta1*^+/+^ littermate controls at 6 months of age (Fig. 6C). Both soluble and insoluble Aβ_1-40_ levels were significantly increased in *Snta1*-null mice (Fig. 6D,E, *P* = 0.0022, *P* = 0.0277, respectively; *t*-test). Soluble Aβ_1-42_ levels were significantly elevated with *Snta1* gene deletion (Fig. 6F, *P* = 0.0111, *t*-test), while the change in insoluble Aβ_1-42_ levels did not reach statistical significance (Fig. 6G, *P* = 0.1395, *t*-test). To investigate which changes in AQP4 localization are most closely related to these increases in Aβ deposition, we evaluated the relationship between Aβ levels and cortical and hippocampal capillary- and large vessel-associated measures of perivascular and astroglial AQP4 IF measured in from the same animals. The complete results of these association studies are shown in Supplemental Figure 14. The most consistent association observed was between declining cortical capillary-associated PV Endfoot AQP4 IF and increasing soluble Aβ_1-40_, Aβ_1-42_, and insoluble Aβ_1-40_ (Fig. 6D–G). Reductions in cortical large vessel-associated perivascular endfoot AQP4 were similarly associated with soluble Aβ_1-40_ and Aβ_1-42_. These associations were restricted to changes in cortical AQP4, as AQP4 IF measures in the hippocampus were not significantly associated with changes in any Aβ measure (Supplemental Figure 14). When a similar association study was undertaken evaluating the relationship between changes in capillary- and large vessel-associated fine process AQP4 expression in the AAV-treated tissue described in Fig. 5 above, no association was observed between soluble or insoluble Aβ_1-40_ or Aβ_1-42_ levels and fine process AQP4 IF either in the cortex or hippocampus (results of the association study are shown in Supplemental Figure 15). These findings suggest that loss of AQP4 from cortical perivascular astroglial endfeet specifically promotes the deposition of Aβ while the distribution of AQP4 to non-perivascular fine processes does not impact Aβ deposition.

## Discussion

Although the slowed clearance of Aβ both with advancing age [3] and the onset of sporadic AD [4] is presumed to contribute to the development of Alzheimer’s pathology and disease progression, the biological changes underlying this slowed clearance and the brain’s vulnerability to such protein mis-aggregation remains unresolved. While AQP4 has been implicated in the perivascular clearance of Aβ, the impact of age- and AD-associated changes in AQP4 localization in the development of Aβ pathology has not been defined. Here we demonstrate that in human AD, astroglial AQP4 localization to perivascular endfoot processes is reduced while localization to fine processes is increased. This loss of perivascular AQP4 localization is associated within increasing local Aβ and tau pathological burden, and with cognitive and functional decline early in the disease process. Our data elaborating on findings in mice [13] confirm that these two phenotypic changes are similarly observed in the aging rodent brain. Using an AAV-based approach to drive AQP4 overexpression, we observe that the distribution of AQP4 to fine non-perivascular processes does not impair perivascular CSF-ISF exchange or alter Aβ deposition in the Tg2576 model of amyloidosis. Using the *Snta1*^−/−^ mouse line, we observe that loss of AQP4 from perivascular astroglial endfoot processes both slows perivascular solute exchange and accelerates Aβ deposition in Tg2576 mice. These findings suggest that age- and brain injury-associated loss of perivascular AQP4 localization may be a key driver in the development of Alzheimer’s pathology and disease progression.

### Reduced perivascular AQP4 localization in human AD

In a prior human post mortem histopathological study, we reported that in the frontal cortex perivascular AQP4 localization declined with age and that this decline associated with dementia diagnosis [20]. The present study extends these prior findings in a substantially larger case series including frontal cortical (*n* = 49) and hippocampal (*n* = 37) samples from subjects over 65 years of age spanning the clinical spectrum from cognitively normal, to MCI, to AD. This case series was developed to directly examine the relationship between changes in AQP4 localization and the development of Alzheimer’s pathology, selecting only cases with a clinico-pathological diagnosis of either “Alzheimer’s disease” or “Alzheimer’s-related pathological changes,” and excluding subjects with significant non-AD pathology. The resulting case series was largely independent from that of our prior study (only 6 overlapping cases). The present study extends prior findings by demonstrating that while subjects with a clinical AD diagnosis exhibited reduced perivascular AQP4 localization compared to cognitively normal individuals, subjects with a clinical diagnosis of MCI exhibited an intermediate AQP4 localization phenotype. In addition, the present study evaluated AQP4 localization in frontal cortical gray matter, frontal cortical white matter, and in the CA1, CA2, and CA3 hippocampal subfields, observing that the association between reduced perivascular AQP4 localization with cognitive status prior to death was specific to changes within the frontal cortical gray matter.

The inclusion of subjects across the spectrum of clinical and pathological AD permitted us to evaluate the association between changes in frontal cortical perivascular AQP4 localization, local measures of Alzheimer’s pathology, and clinical and functional decline in detail. Our findings from the present rodent studies demonstrate that changes in perivascular AQP4 localization are sufficient to impair CSF-ISF exchange and promote Aβ deposition. Aβ deposition begins early in the AD pathological cascade, while Aβ burden plateaus in the course of clinical progression [38–41]. The present observation that among cognitively intact and MCI subjects reduced perivascular AQP4 localization was associated with higher CERAD neuritic plaque scores is consistent with a role for changes in AQP4 localization in human amyloid β deposition.

Similarly, AQP4-dependent glymphatic exchange has been implicated in the clearance of tau, and the impairment of this perivascular exchange has been proposed to account in part to the development of age-related and post-traumatic tauopathy [14, 42]. When we measured local pathological tau burden within the frontal cortex, we observed that as with amyloid β pathology, declining perivascular AQP4 localization was associated with increased p-tau burden in non-demented subjects. These findings were paralleled by associations between reduced perivascular AQP4 localization and lower scores on measures of cognitive and functional status, the CDR SoB. The fact that these associations were maintained only in non-demented subjects, and not in subjects with clinical AD diagnosis, argues that changes in AQP4 localization may be exerting their effect early in the AD pathological process, rather than in later stages of the disease process. Our observation that the association with CDR SoB score and with local measures of AD-related neuropathology remained significant even when controlling for cognitive status using partial correlation analysis argues that these effects do not simply reflect differences in neuropathology across non-demented groups.

One key limitation of any post mortem histopathological study is the inability to ascertain causality. The association of changes in perivascular AQP4 localization with measures of pathological and clinical AD progression may result from the activation of cortical astrocytes associated with the development of amyloid β plaques. The present experimental studies in rodents demonstrate that such changes in AQP4 localization are sufficient to promote Aβ pathology. However, they do not confirm that this actually occurs in the setting of human disease. Genetic association studies may provide important insight into the role that perivascular astroglial biology may play in the development of AD in human populations. For example, we reported in a limited candidate gene-association study that five naturally occurring single-nucleotide polymorphisms (SNPs) in the human *AQP4* gene are associated with cognitive impairment in the setting of AD [43]. In two subsequent studies, SNPs in the *AQP4* gene were observed to moderate the relationship between sleep disruption and amyloid β pathology [44] and to be associated with variation in amyloid β burden and clinical AD progression [45]. These studies, while preliminary, support a causal link between AQP4 and the development of AD pathology and AD clinical progression. No study to date has evaluated whether SNPs in the genes whose products determine perivascular AQP4 localization, such as a-syntrophin (*SNTA1*)*,* dystrobrevin (*DTNA*), or dystroglycan (*DAG1*) are associated with variation in AD pathology or progression. This question remains an important subject for future research.

### The role of AQP4 in perivascular CSF-ISF exchange

In the initial description of the glymphatic system, perivascular CSF-ISF exchange and interstitial Aβ clearance were observed to be impaired by *Aqp4* gene deletion [11], suggesting a key role for glial water transport in perivascular fluid and solute movement. Although one study failed to replicate this observation [46], a subsequent study including findings from five different labs and using five independent transgenic mouse lines [17] confirmed the initially observed role of AQP4 in perivascular CSF-ISF exchange. More recently, studies utilizing an AQP4-specific inhibitor TGN-02 0[47, 48] report that acute inhibition of AQP4 slows perivascular exchange and Aβ clearance [42, 49]. These findings are corroborated by studies demonstrating that *Aqp4* gene deletion in mouse models of amyloidosis accelerates Aβ deposition [18, 50].

Our prior findings show an association between loss of perivascular AQP4 localization and impairment in glymphatic function in the aging, post-traumatic, and ischemic brain [13–15], yet these studies did not directly test whether this loss of perivascular localization reduces CSF-ISF exchange or alters the development of AD pathology. The present observation that *Snta1* gene deletion, which abolishes cortical perivascular AQP4 localization, disrupts CSF tracer influx and interstitial solute efflux while increasing Aβ deposition supports the overall role of AQP4 in perivascular CSF-ISF exchange. It also provides the first causal evidence that loss of perivascular AQP4 localization impairs CSF-ISF exchange and can promote neurodegenerative pathology. It is important to note that the loss of perivascular AQP4 localization in the *Snta1*^−/−^ mouse model is more pronounced than that observed in the post mortem cortex in the setting of human aging and AD. Unlike the development of Aβ and tau pathology in transgenic mouse lines that occurs over the course of months, the pathological accumulation of Aβ and tau aggregates in the human brain occurs over the course of years prior to the onset of AD-related clinical symptoms [40]. In addition, the anatomical distances for perivascular Aβ clearance in the human brain are much greater than those in the rodent brain. These differences in anatomical scale may permit relatively subtle changes in AQP4 localization to contribute to the development of neuropathology in the human brain over the extended timescale observed in the setting of human AD.

Whether changes in SNTA1 expression, or changes in the expression of other elements of the astroglial dystrophin-associated complex underlie age- and injury-associated changes in AQP4 localization has not yet been defined. Indeed, recent transcriptomic profiling studies from mice and human brain tissue have identified several potential perivascular endfoot elements whose expression profiles in development and the setting of AD parallel that of AQP4. Expression levels of these elements predict dementia status and p-tau levels in the aging human brain [51–53] and dystrophin-deficient astrocytes have altered astrocyte function and altered AQP4 levels [54]. Whether these putative endfoot elements contribute to changes in AQP4 localization, perivascular CSF-ISF exchange, or the development of AD pathology remains unknown. Similarly, whether SNTA1 expression regulates their localization or function has not been evaluated.

It is noteworthy that the reduction in perivascular CSF-ISF exchange described in the present study is more modest than observed in *Aqp4*^−/−^ mice [11, 14, 17, 37]. For example, differences in CSF tracer distribution between wild type and *Snta1*^−/−^ mice were evident 90 min, but not 30 min following intracisternal injection, whereas differences between *Aqp4*^−*/*−^ and wild type mice are readily observed at the 30 min time point. We also observed regional, rather than global changes in CSF tracer influx in the *Snta1* knockout line. If AQP4 supports perivascular CSF-ISF exchange, then it is not surprising that a genetic model lacking perivascular localization yet preserving overall AQP4 expression exhibits a regional phenotype relative to a global knockout. The regional changes in CSF tracer influx observed in the hippocampus and diencephalon following *Snta1* gene deletion in Tg2576 mice may have important implications for AD. In AD, both Aβ and p-tau pathology arise earliest in specific neuroanatomical locations, with pathology propagating or spreading to other involved regions over time. The hippocampus is an early site of AD pathology [55] and plays a crucial role in memory formation [56]. Thus, the observed vulnerability of hippocampal glymphatic clearance to changes in AQP4 localization in rodents may reflect the role that local differences in baseline rates of glymphatic clearance or in age- and injury-associated impairment in glymphatic function may play in the neuroanatomical distribution of AD-related pathology.

### The role of AQP4 isoforms in astroglial localization

In mouse models of aging [13], traumatic brain injury [14, 21], and ischemic injury [15, 22], the loss of AQP4 from perivascular endfoot domains is accompanied by distribution of AQP4 to fine non-perivascular processes. In the setting of aging and AD, present and prior [20] studies in post mortem human tissue demonstrate the emergence of a dispersed, cortical astrocyte population with intense AQP4 immunoreactivity spread throughout its fine processes which underlies the development of a “patchy” pattern of AQP4-IF throughout the cortical depth. The biological rationale and trigger for this upregulation remain unclear. One possibility is that blood-brain barrier disruption, a process that occurs in aging and neurodegeneration [9, 57], promotes reactive astrogliosis and upregulation of AQP4. It is possible that impaired perivascular clearance resulting from astrogliosis and the loss of perivascular localization occur in parallel with blood-brain barrier disruption, with both processes contributing to slowed Aβ clearance. It is also possible that waste accumulation at the perivascular compartment due to loss of perivascular AQP4 localization is a precursor to widespread cerebrovascular neuroinflammation and downstream blood-brain barrier disruption. Clearly, further investigation into the interrelationship between these processes is warranted.

In one arm of the present study, we tested whether the distribution of AQP4 *to* astroglial fine processes, rather than its removal *from* perivascular endfoot processes, could impair perivascular CSF-ISF exchange and alter Aβ deposition. In agreement with prior in vitro studies [32], we observed that the AQP4-M1 and AQP4-M23 isoforms exhibit distinct subcellular localization profiles when expressed both in vitro and in vivo. AQP4-M1 localized to small puncta dispersed over the fine processes of astrocytes while AQP4-M23 clustered into larger puncta that concentrated around perivascular astroglial endfeet. This is consistent with previous reports that suggest that palmitoylation sites specific to the NH_2_ terminus of the AQP4-M1 isoform inhibit its assembly into orthogonal arrays and localization to perivascular endfoot domains [58]. These results suggest that the differential expression of AQP4-M1 and AQP4-M23 isoforms in vivo may contribute both to the localization of AQP4 to perivascular astroglial endfeet as well as to the localization of AQP4 to fine non-perivascular processes under pathological conditions.

The viral overexpression of AQP4-M1 resulted in AQP4 localization similar to the distribution to fine non-perivascular astroglial processes observed both in the aging rodent and human AD brain. We observed that neither the overexpression of AQP4-M1 nor of AQP4-M23 altered either perivascular exchange or Aβ deposition. Indeed, while variation in cortical perivascular endfoot localization of AQP4 was significantly associated with Aβ burden among Tg2576:*Snta1*^−/−^ and Tg2576:*Snta1*^+/+^ mice, no significant associations between non-perivascular AQP4 localization and Aβ burden were observed with AQP4-M1 or AQP4-M23 overexpression. These results, along with those from the *Snta1*^−/−^ mice, suggest that it is the loss of AQP4 *from* perivascular astroglial endfoot domains, and not the distribution of AQP4 *to* non-perivascular fine astroglial processes that impairs CSF-ISF exchange and the clearance of Aβ in the aging brain.

A recent study demonstrated that AQP4 localization is regulated dynamically, undergoing changes in subcellular localization an plasma membrane insertion over a much shorter timescale than previously appreciated [59–61]. Whether such dynamic regulation of subcellular AQP4 localization is altered in the setting of aging or AD, and whether such changes impair glymphatic function or contribute to the development of AD pathology remains an important subject for future inquiry. In addition, two independent groups have reported that AQP4 exhibits an exceptionally high rate of translational readthrough, resulting in the common occurrence of a novel isoform AQP4-X (also termed AQP4-ex) [62, 63]. This variant exhibits robust perivascular localization and is less dramatically upregulated in response to reactive astrogliosis than other AQP4 isoforms [63]. While the present study suggests that upregulation of AQP4-M1 and AQP4-M23 likely do not account for impairment of perivascular exchange and Aβ clearance in the aging brain, whether changes in AQP4-X expression or localization regulate perivascular clearance mechanisms, remain an important, unanswered question. Although it is reasonable to suppose that perivascular AQP4-X localization is dependent on SNTA1 expression, this has never been directly tested.

## Conclusion

These data suggests that AQP4 in perivascular astroglial endfoot processes plays a vital role in facilitating the influx and efflux of proteins in and out of the brain, and that disruption of perivascular AQP4 localization, such as occurs in the aging and injured brain, can promote the development of Aβ pathology. The observation that loss of perivascular AQP4 localization in post mortem human tissue is a feature of AD and associates with both Alzheimer’s pathology and cognitive/functional decline early in the disease process suggest that these mechanisms may be key drivers in the development and progression of Alzheimer’s disease in human populations.

### Limitations

Several open questions remain regarding the fundamental mechanisms of glymphatic function, perivascular CSF-ISF exchange, and their role in the pathogenesis of AD. It is currently unclear precisely how AQP4 supports macroscopic perivascular CSF-ISF exchange throughout the brain. While computational studies of CNS fluid dynamics on the microscopic (cellular) scale have failed to fully explain the role of AQP4 in perivascular exchange [18, 63], findings from an increasing number of studies carried out in *Aqp4*^−/−^ [11, 14, 17, 37], in *Snta1*^−/−^ mice ([17], present findings) and with AQP4 inhibitors [42] clearly demonstrate a critical role for perivascular AQP4 in CSF-ISF exchange. A second, related limitation is the fact that our analysis of perivascular AQP4 localization in both the human and rodent brain did not distinguish between localization surrounding arterial versus venous segments. The initial characterization of the glymphatic system described the fact that perivascular AQP4 localization was greater surrounding cortical ascending veins than around penetrating arteries (Supplementary Figure 7 in [11]), a pattern of localization that may contribute to the organization of fluid and solute movement through brain tissue. In the present study, neither AQP4-M1 overexpression nor *Snta1* gene deletion preferentially targeted artery versus vein-associated perivascular astrocytes. Thus, it cannot shed light on the role that vascular segmental differences in AQP4 localization play in the supporting perivascular exchange and Aβ clearance. Furthermore, our study examined CSF-ISF exchange and Aβ levels in 6-month-old Tg2576 following manipulation of AQP4 localization via either AAV treatment or when crossed with Snta1 KO mice. This 6-month timepoint was chosen in order to be able to detect accelerated progression of Aβ pathology in these mice and to avoid a possible ceiling effect in Aβ levels in older Tg2576 mice. However, similar experiments conducted in further aged mice may show a similar phenotype. Similar results were seen in the APP/PS1 line when crossed with AQP4 KO at 12 months of ages, as published by Xu et al. [18]. There are several limitations to our AAV approach; given that we administered our AAVs intravenously via the retro-orbital sinus, we cannot rule out the possibility that the transduction efficiency around capillaries may have been higher than for large vessels and the wider neuropil. In addition, intravenously administered AAV-PHP viruses have been reported to be more efficient at transducing astrocytes in female mice [64]. Both sexes were represented equally in all groups of mice and obvious stratification was not seen within virus injected groups for any of the analyses; however, the AAV transduction efficiency could have been more efficient in our female mice.

## Supplementary Information

**Additional file 1.**

## Data Availability

The datasets used and/or analyzed during the current study are available from the corresponding author on reasonable request.

## Abbreviations

*Snta1*: α-Syntrophin
AAV: Adeno-associated virus
AD: Alzheimer’s disease
Aβ: Amyloid β
ANOVAs: Analysis of variance
AQP4: Aquaporin-4
AALAC: Association for Assessment and Accreditation of Laboratory Animal Care
BiG: Binary GaAsP
BSA: Bovine serum albumin
CDR SoB: CDR Sum of Boxes
CSF: Cerebrospinal fluid
CDR: Clinical Dementia Rating
CN: Cognitively normal
CERAD: Consortium to Establish a Registry for Alzheimer’s Disease
*DTNA*: Dystrobrevin
*DAG1*: Dystroglycan
eGFP: Enhanced green fluorescent protein
ELISA: Enzyme-linked immunosorbent assay
HA: Hemagglutinin
IF: Immunofluorescence
IACUC: Institutional animal care and use committee
IR: Intra-quartile range
KX: Ketamine/xylazine
MCI: Mild cognitive impairment
MMSE: Mini-Mental State Examination
OHSU: Oregon Health and Science University
PV Endfoot: Perivascular endfoot
ROIs: Regions of interest
SNPs: Single-nucleotide polymorphisms
WPRE: 3′ Woodchuck hepatitis virus posttranscriptional regulatory element
