# Supplementary material for: Loss of perivascular aquaporin-4 localization impairs glymphatic exchange and promotes amyloid β plaque formation in mice
Source: Alzheimers Res Ther. 2022 Apr 26;14:59. doi: 10.1186/s13195-022-00999-5 (PMC9040291; doi:10.1186/s13195-022-00999-5)
Supplement: Supplementary file 1 — Additional file 1. [file 13195_2022_999_MOESM1_ESM.docx]

**Supplemental Materials**

**Supplemental Tables**

**Supplemental Table 1. AAV construct summary**

| **Abbreviated Name** | **Full-Length Name** | **Serotype** | **Protein** | **Isoform** | **Promoter** | **Tag** | **P2A Site** | **Co-Expressed** |
| --- | --- | --- | --- | --- | --- | --- | --- | --- |
| AAV8-M1 | AAV.8-GfaABC1D-AQP4-M1-eGFP | AAV.8 | AQP4 | M1 | GfaABC1D | eGFP | No | N/A |
| AAV8-M23 | AAV.8-GfaABC1D-AQP4-M23-eGFP | AAV.8 | AQP4 | M23 | GfaABC1D | eGFP | No | N/A |
| AAV^PHP^-GFP | AAV.PHP.B-GfaABC1D-GFP | AAV.PHP.B | eGFP | N/A | GfaABC1D | N/A | No | N/A |
| AAV^PHP^-M1 | AAV.PHP.B-GfaABC1D-AQP4-M1 | AAV.PHP.B | AQP4 | M1 | GfaABC1D | N/A | No | N/A |
| AAV^PHP^-M23 | AAV.PHP.B-GfaABC1D-AQP4-M23 | AAV.PHP.B | AQP4 | M23 | GfaABC1D | N/A | No | N/A |
| AAV^PHP^-M1-HA | AAV.PHP.B-GfaABC1D-AQP4-M1-HA-P2A-eGFP | AAV.PHP.B | AQP4 | M1 | GfaABC1D | HA | Yes | eGFP |
| AAV^PHP^-M23-HA | AAV.PHP.B-GfaABC1D-AQP4-M23-HA-P2A-eGFP | AAV.PHP.B | AQP4 | M23 | GfaABC1D | HA | Yes | eGFP |

**Supplemental Figures**


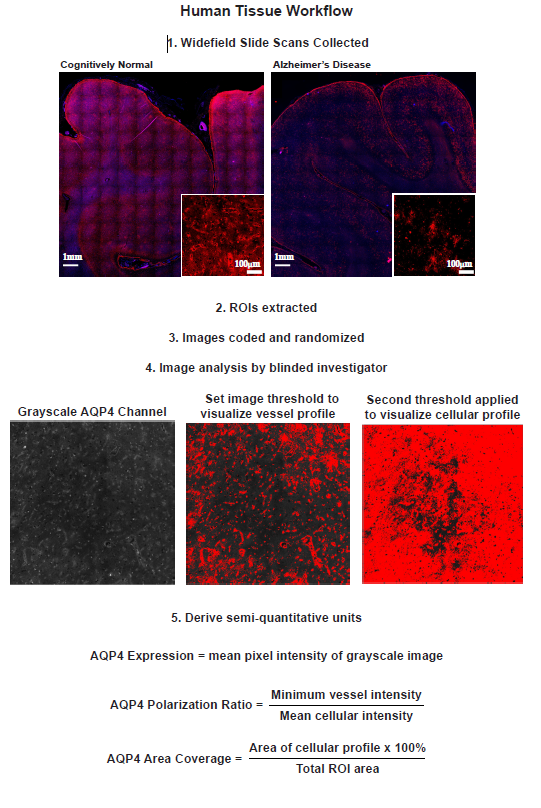


**Supplemental Figure 1. Human tissue AQP4 immunofluorescence analysis workflow.** (**1**) The frontal cortical tissues of CN, MCI, and AD subjects were immunolabeled with an antibody against AQP4 and imaged using a ZEISS AxioScan widefield slide scanner. (**2**) Multiple ROIs were extracted from the original images. (**3**) Images were coded with a random number and (**4**) analyzed by a blinded investigator. To quantify AQP4 immunofluorescence in different astroglial compartments, the fluorescence images of AQP4 channel were converted into grayscale, then a threshold was set to visualize the vessel profiles. A second threshold was then set to visualize the cellular profiles of AQP4 immunofluorescence. (**5**): AQP4 expression, polarization ratio, and area coverage each quantified from these measured values.


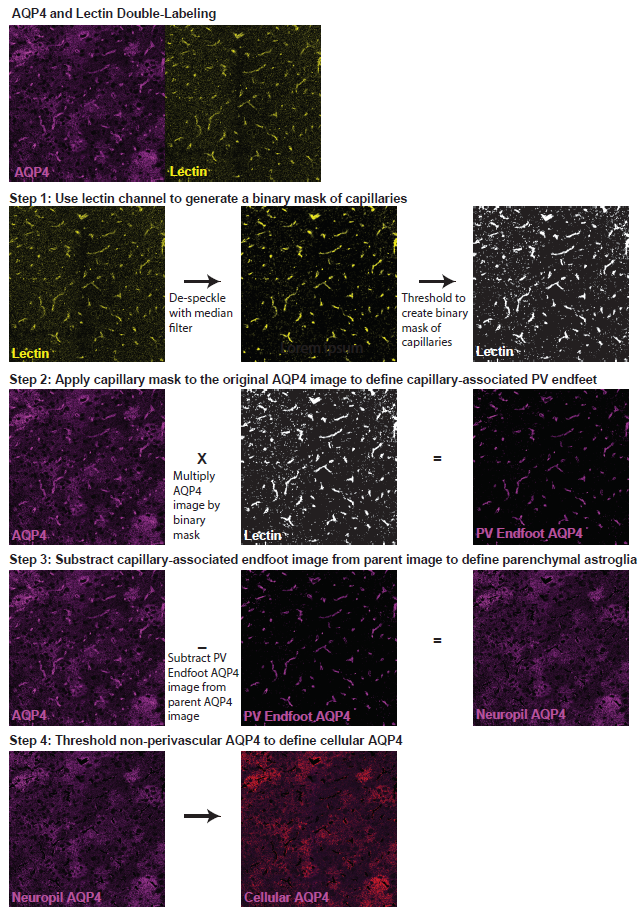


**Supplemental Figure 2. AQP4 IF analysis workflow in capillary-associated astrocytes.** Mouse brain sections were co-stained with anti-AQP4 antibody and lectin, and analyzed in ImageJ. **Step 1.** To quantify AQP4 signal in capillary-associated perivascular astrocytic endfeet (PV Endfeet) , ROIs were selected in cortex and hippocampus to avoid large vessels. The lectin channel was used to generate a capillary mask by applying a median filter to de-speckle the image, and by setting a threshold to generate a binary capillary mask. **Step 2.** By multiplying the original AQP4 image and the binary capillary mask image, the PV Endfoot AQP4 image was generated and the PV Endfoot AQP4 IF was quantified. **Step 3.** To generate the non-perivascular neuropil AQP4 image, the PV Endfoot AQP4 image was subtracted from the parent AQP4 image and neuropil AQP4 IF was measured. **Step 4.** To calculate the cellular coverage of AQP4, the non-perivascular neuropil AQP4 image was thresholded to highlight cellular AQP4 labeling, and the AQP4 area coverage was calculated.

**
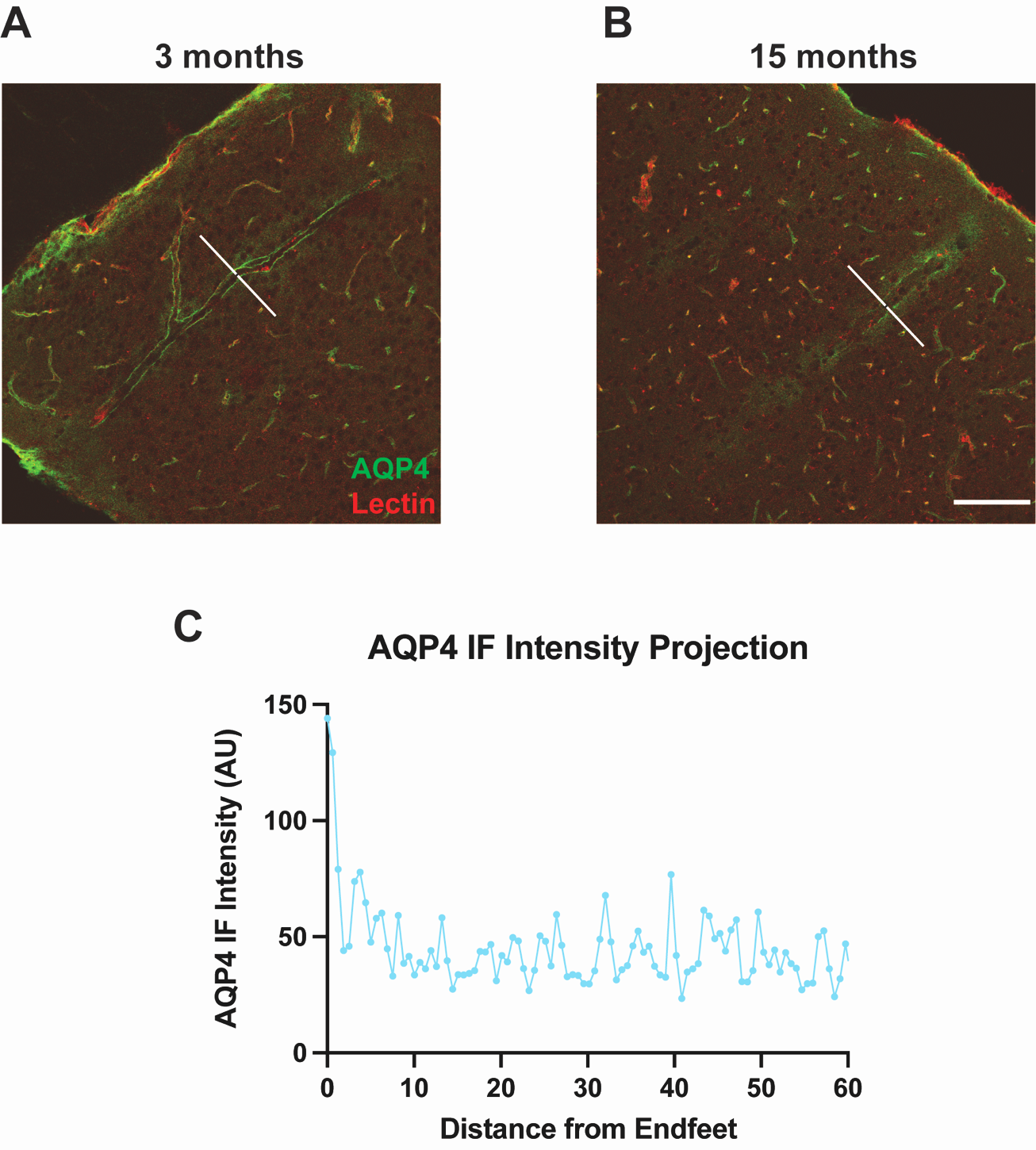
**

**Supplemental Figure 3. AQP4 immunofluorescence analysis workflow for large vessels.** Mouse brain sections were co-stained with anti-AQP4 antibody and lectin and analyzed in ImageJ. For AQP4 IF analysis, vessel diameter was measured and lines were drawn, on both sides, through the perivascular endfoot, continuing through the surrounding astrocytes and neuropil. Both intensity projection plots were averaged for a single intensity plot per vessel and then were averaged across groups. For graphing of segments, pixel intensities were binned and averaged across these line segments. The first 5 pixels were averaged for the perivascular endfoot segment, the next 30 pixels were averaged for the astrocyte segment, and the last 80 pixels were averaged for the neuropil segment. (**A**) Representative image of AQP4 and lectin staining in a 3m old and (**B**) 15 month old wild-type mouse brain cortex. Scalebar = 100 um. Example lines for intensity projections are shown. (**C**) Example representative intensity projection plot for single large vessel from 3 month-old mouse cortex.


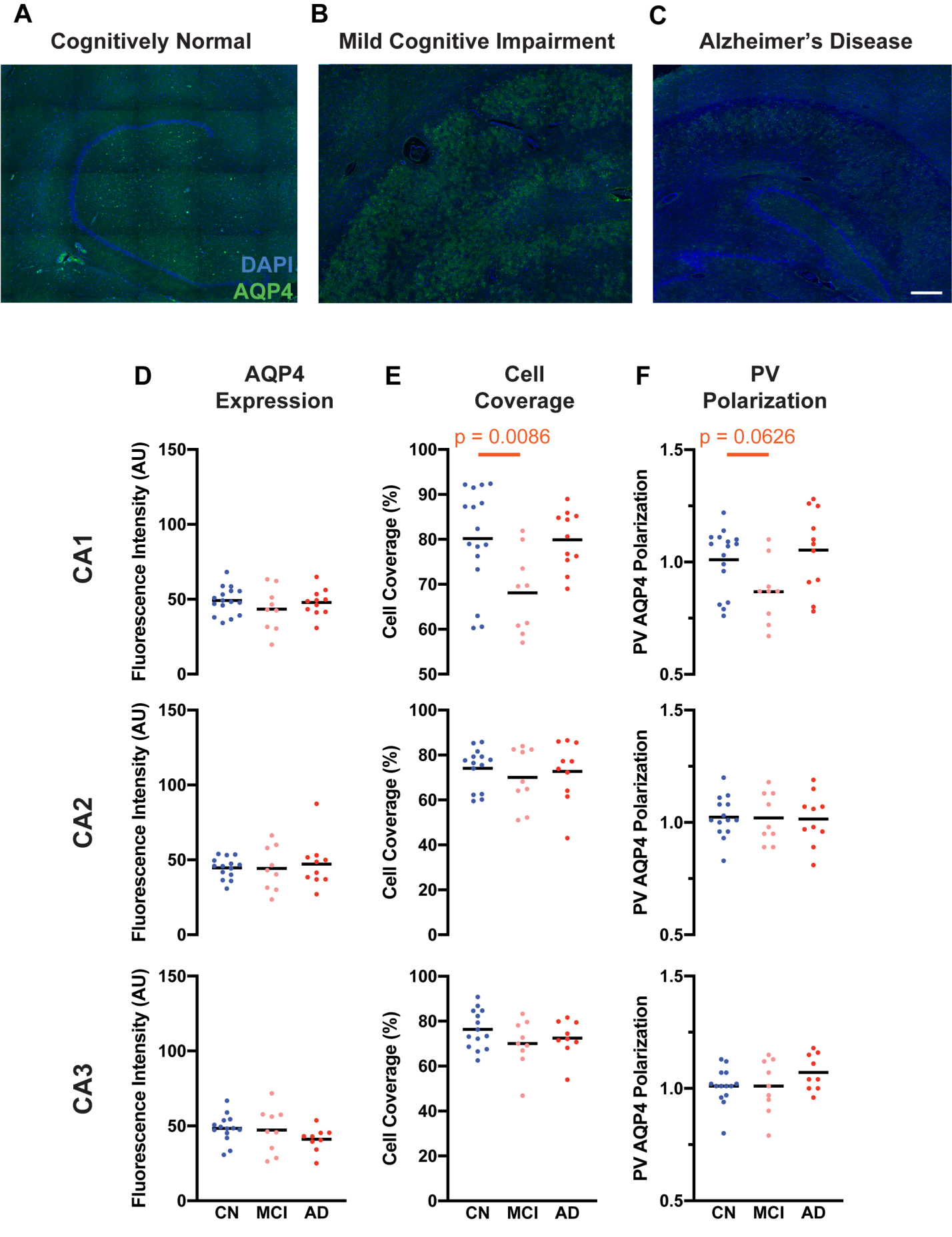


**Supplemental Figure 4. AQP4 expression and polarization in human hippocampus**

(A) Representative images of the immunofluorescent labeling of AQP4 and DAPI labeling in hippocampus of cognitively normal (**A**), mild cognitive impairment (**B**), and Alzheimer’s disease (**C**) subjects. Scale bar = 500 µm. (**D**) The AQP4 IF in the hippocampal CA1, CA2, and CA3 regions were comparable between CN, MCI, and AD subjects. (**E**) The AQP4 cell coverage in the hippocampal CA2 and CA3 regions were comparable between CN, MCI and AD subjects, while in the CA1 region was significantly reduced in MCI subjects compared to the CN subjects (P = 0.0086, One-Way ANOVA with Dunnett’s post hoc correction). (**F**) The perivascular AQP4 polarization in the hippocampal CA2 and CA3 regions were comparable between CN, MCI and AD subjects, but tended to be reduced in the CA1 region of MCI subjects comparing to the CN subjects (P = 0.0626, One-Way ANOVA with Dunnett’s post hoc correction).

**
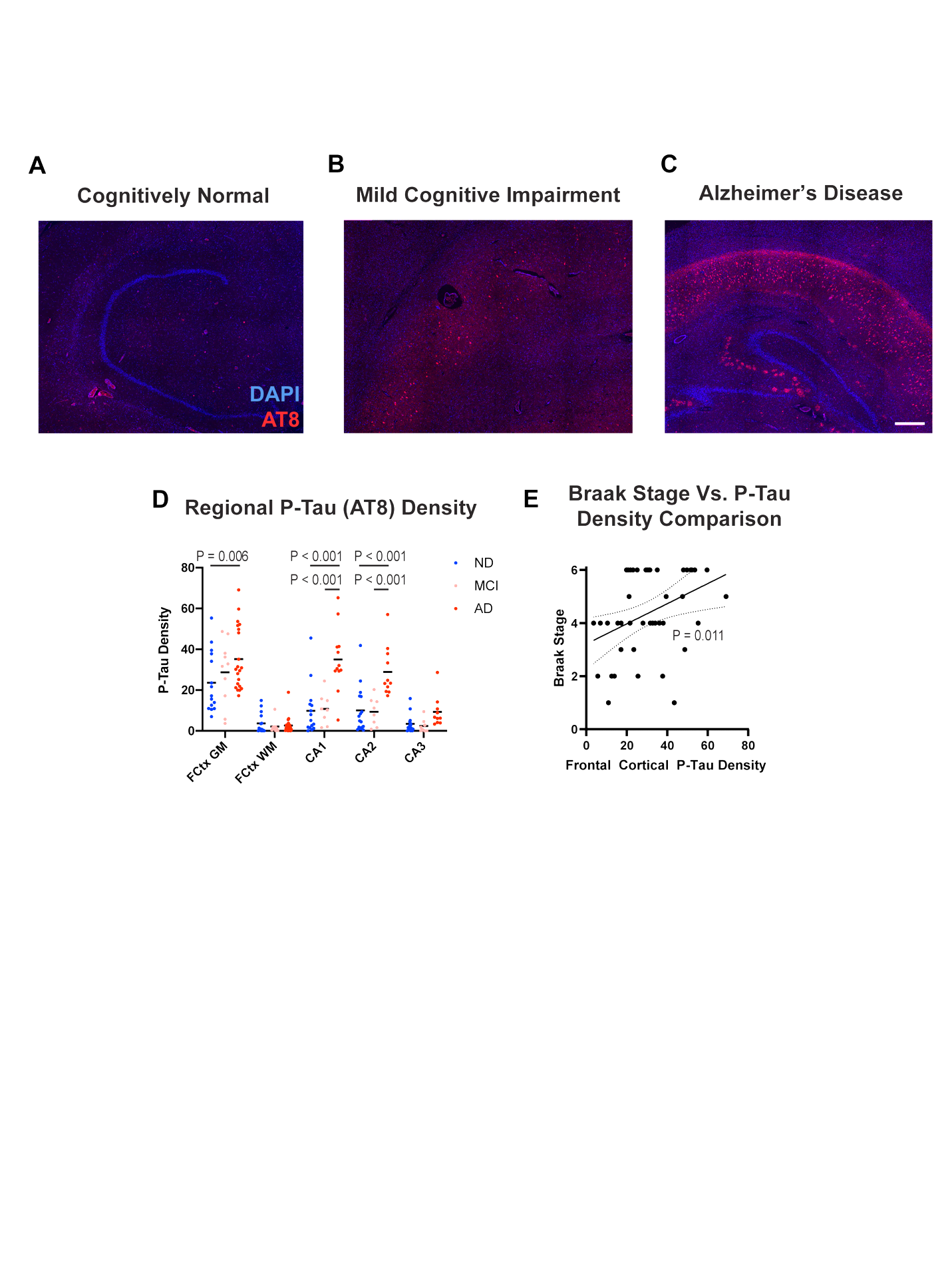
**

**Supplemental Figure 5. Association between p-tau and Braak stage in CN, MCI and AD subjects.**  Representative images of the immunofluorescent labeling of AT8-labeled p-tau and DAPI in hippocampus of cognitively normal (**A**), mild cognitive impairment (**B**), and Alzheimer’s disease **C**) subjects. Scale bar = 500 µm. (**D**) AT8-labeled p-tau IF was significantly increased in the frontal cortical grey matter (P = 0.005, 2-way ANOVA with Tukey’s post hoc test), hippocampal CA1 (P<0.001, 2-way ANOVA with Tukey’s post hoc test) and CA2 (P<0.001, 2-way ANOVA with Tukey’s post hoc test) regions in AD subjects. (E) Braak stage was positively correlated with the frontal cortical p-tau IF (P = 0.011, R^2^ = 0.139).


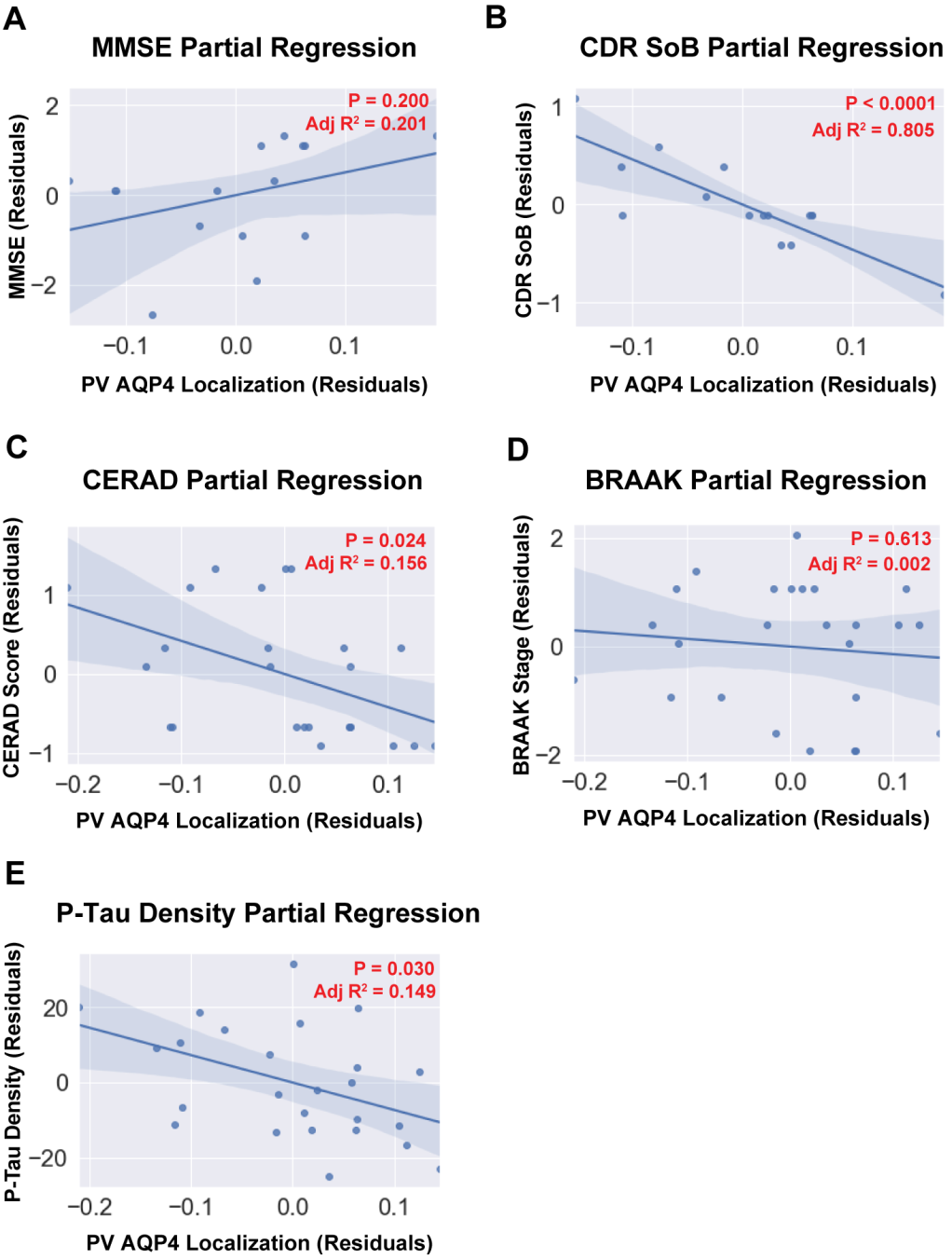


**Supplemental Figure 6. Partial regression plots relating perivascular AQP4 localization with cognitive decline and Alzheimer’s disease associated neuropathology.** Partial regression plots of residuals generated from regression analyses of pooled data from cognitively intact and mild cognitive impairment (MCI) subjects (cognitive status as covariate). (A) Following partial regression analysis controlling for cognitive status, lower perivascular localization was no longer associated with lower Mini Mental State Exam (MMSE) score (P=0.200, Adjusted (Adj) R^2^=0.201); however, (B) higher CDR Sum of Boxes (CDR SoB) score remained associated with perivascular localization (P< 0.0001, Adj R^2^=0.805). (C) Perivascular localization also remained associated with cortical amyloid β plaque density measured by CERAD neuritic plaque score (P=0.024, Adj R^2^=0.156). (D) No association was observed between perivascular AQP4 localization and BRAAK stage (P=.613, Adj R^2^=0.002); however, (E) increased p-tau density in the frontal cortex remained associated with decreased perivascular AQP4 localization (P=0.030, Adj R^2^=0.149).


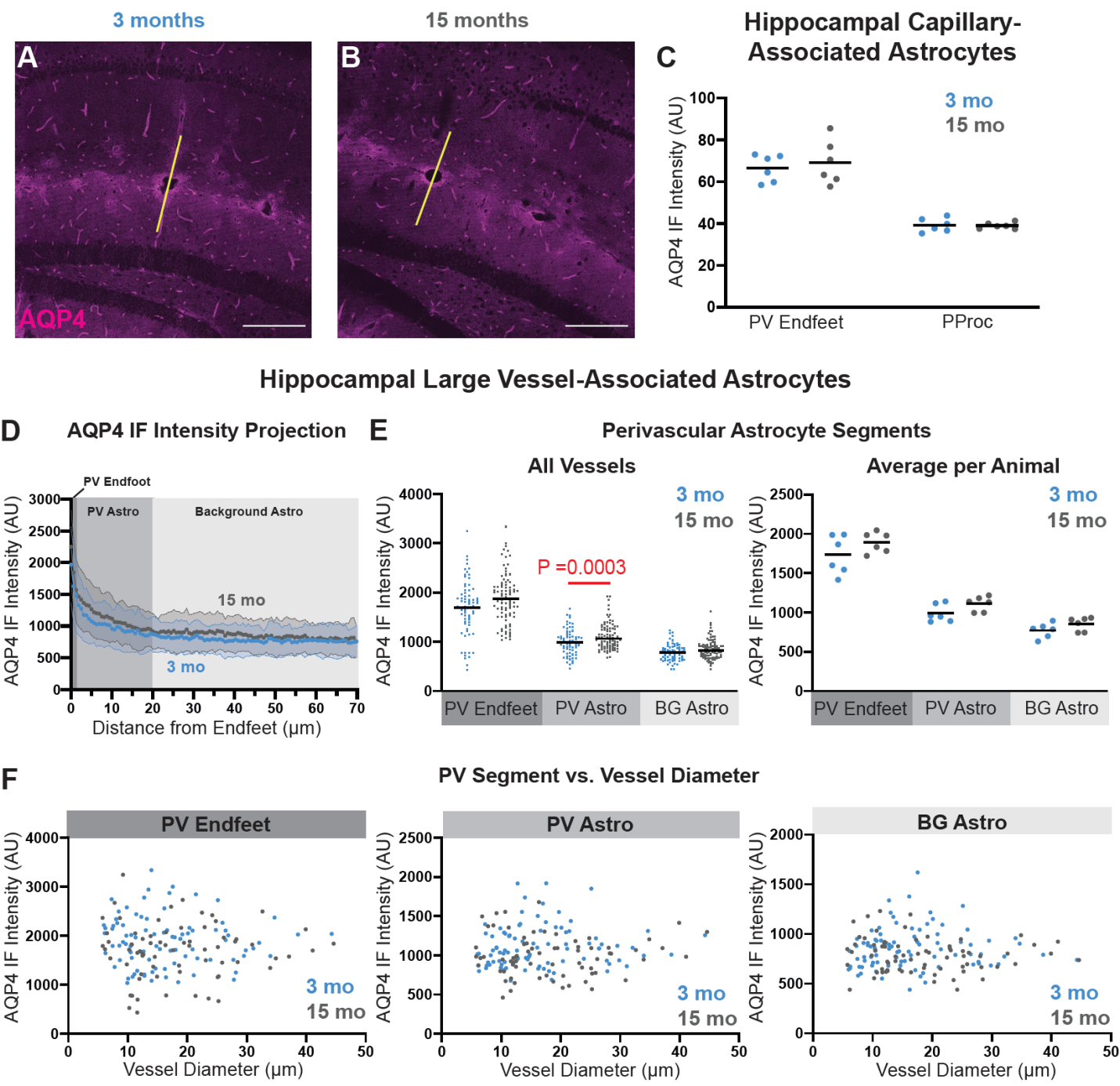


**Supplemental Figure 7. Perivascular AQP4 localization is not altered in the aged mouse hippocampus.** (**A**-**B**): Representative images of AQP4 IF labeling in 3 month (**A**) and 15 month (**B**) mouse hippocampus. Scale bars: 200 µm. (**C**) In capillary-associated astrocytes, neither PV Endfoot AQP4 IF nor non-perivascular neuropil AQP4 IF were altered in aged compared to young mice. (**D**) Cross-sectional AQP4 IF (white lines in (**A**) and (**B**)) at large vessels of the CA1 stratum lacunosum moleculare was evaluated in 3 month (blue; 71 vessels from 6 animals) and 15 month (grey; 89 vessels from 6 animals) old mice. (**E**) When cross-section projections were segmented into PV Endfoot (0-1.5 µm from the vessel wall), PV Astro (1.5-20 µm from the vessel wall), and non-perivascular neuropil (20-68 µm from the vessel wall) segments neither PV Endfoot nor neuropil AQP4 IF differed between young and aged animals. However PV Astro AQP4 IF was significantly higher in the aged mice (P = 0.0003, Mixed effect model with Sidak post hoc correction, statistical tests using all individual vessel values). Plot at left shows individual vessels, plot at right shows values averaged within animals. (**F**) Along hippocampal large vessels, no association was observed between vessel diameter and PV Endfoot (left), PV Astro (middle) or neuropil AQP4 IR (right).

**
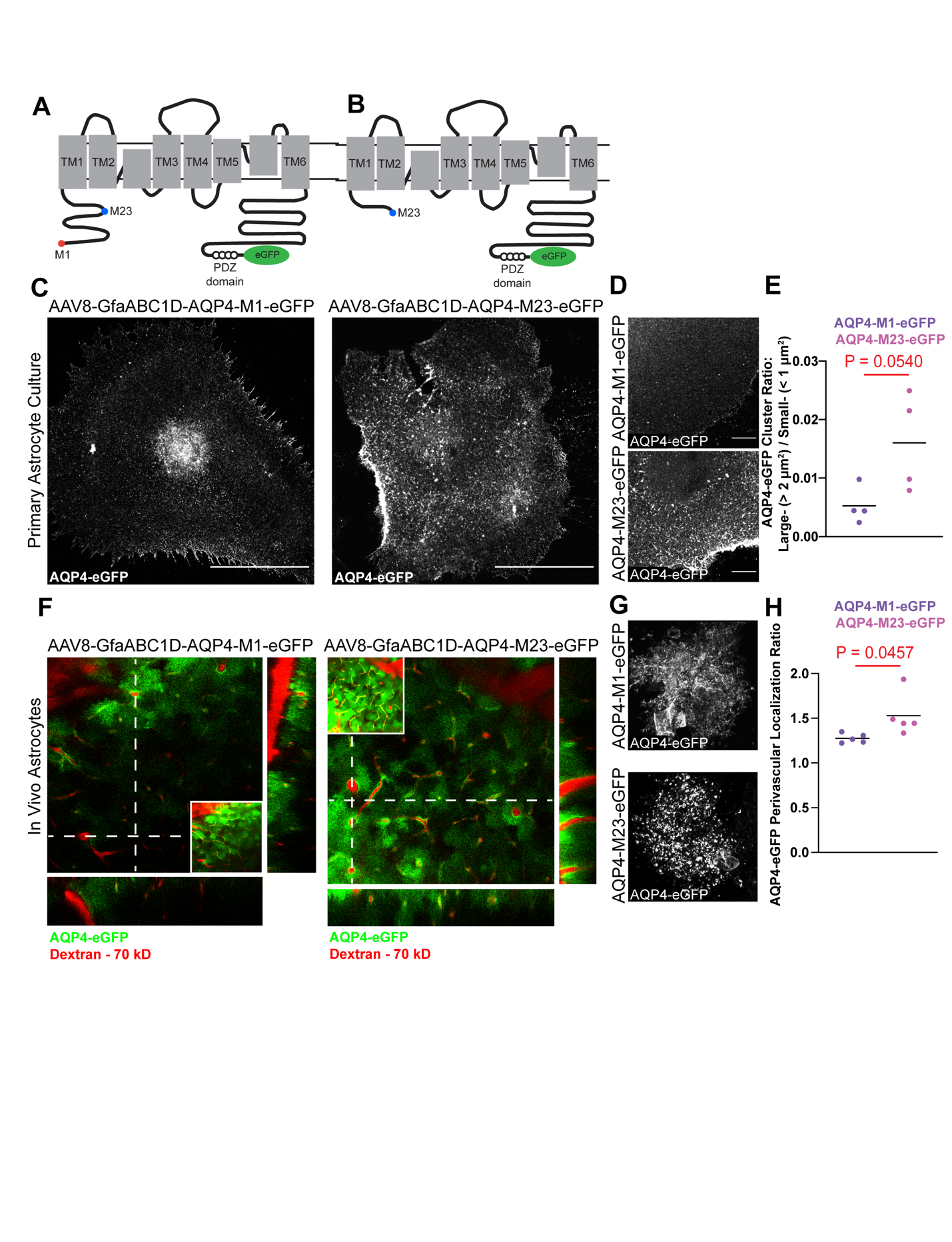
**

**Supplementary Figure 8. AAV8-AQP4-M1/M23-eGFP expression *in vitro* and *in vivo*.**  (**A**-**B**) Schematic outline of the viral approach to overexpress AQP4-M1-eGFP (**A**) or AQP4-M23-eGFP (**B**) isoforms under the astrocyte-specific GfaABC1D promoter in culture and *in vivo*. eGFP was linked to the AQP4 isoforms after the PDZ domain. (**C**) AAV8-M1-eGFP (left) and AAV8-M23-eGFP (right) overexpression in cultured primary cortical astrocytes. Overexpression of AQP4-M23-eGFP resulted in larger fluorescence puncta compared to AQP4-M1-eGFP. Scale bar: 5 µm. (**D**) Higher magnification images of AQP4-M1-eGFP (top) and AQP4-M23-eGFP (bottom) overexpression in cultured primary cortical astrocytes. Scale bar: 1 µm. (**E**) Puncta sizes were quantified and AQP4-M23-eGFP overexpression increased the ratio of large puncta (>2 µm^2^) relative to small puncta (<1 µm^2^) than what was observed in AQP4-M1-eGFP overexpressing cells (P = 0.0540, unpaired t-test). (**F**) AAV8- M1-eGFP (left) and AAV8-M23-eGFP (right) expression *in vivo*. Following the intra-cortical injection of AAV8-M1/M23-eGFP, 2-photon microscopy was used to evaluate the perivascular localization of AQP4-M1/M23-eGFP in reference to the cerebral vasculature labeled with an intravascular fluorescent tracer (70 kD Texas Red-conjugated dextran). Both AAV8-M1-eGFP and AAV8-M23-eGFP injection resulted in widespread cortical astroglial AQP4-eGFP expression. (**G**) Visualization of individual astrocytes showed that AQP4-M1-eGFP (top) overexpression resulted in small fluorescence puncta dispersed throughout astroglial fine processes, while AQP4-M23-eGFP (bottom) puncta were larger in size, which was consistent with observation *in vitro*. (**H**) AQP4-M23-eGFP expression was significantly more perivascular in its localization than AQP4-M1-eGFP *in vivo* (P = 0.0457, unpaired t-test).

**
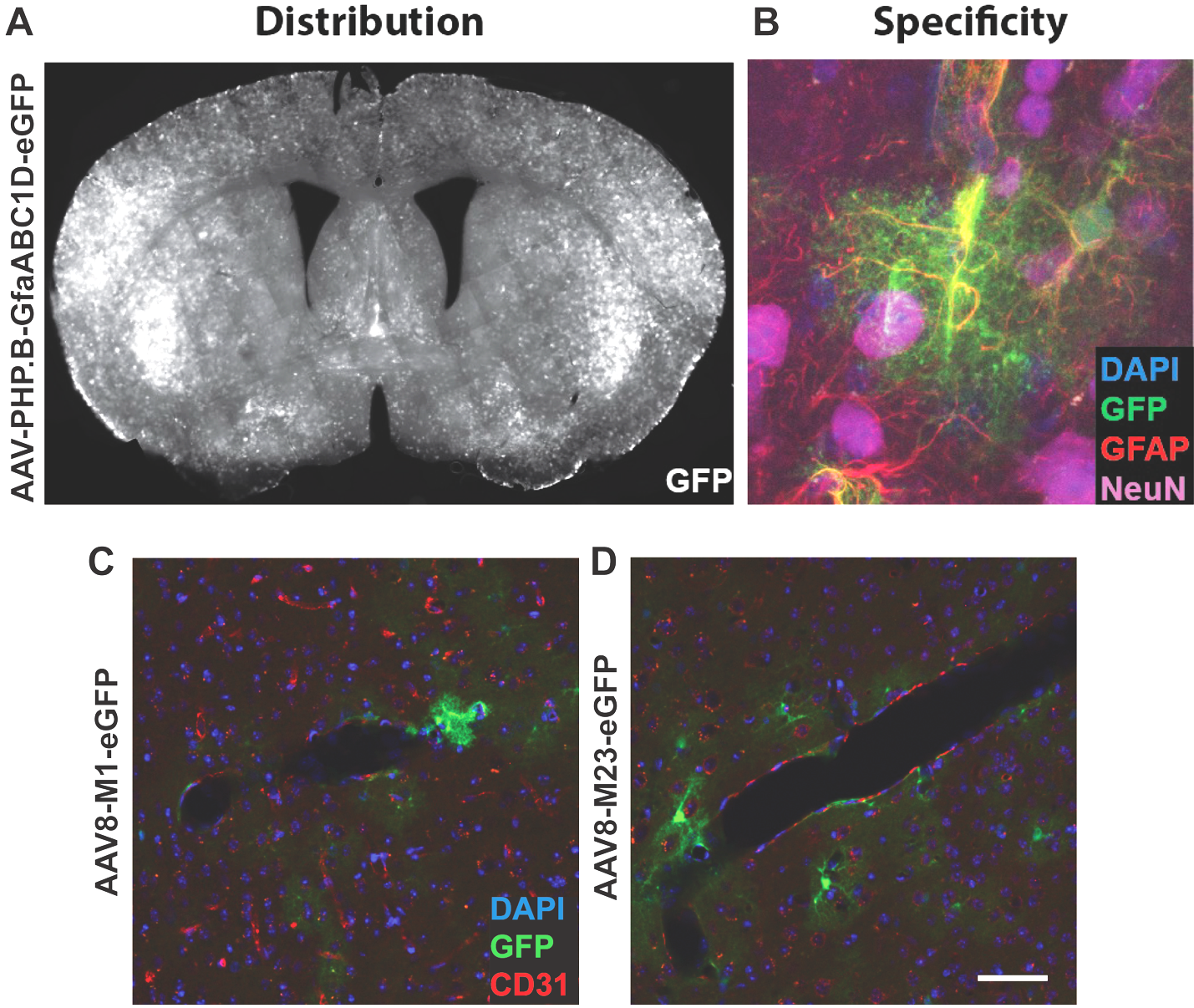
**

**Supplemental Figure 9. Distribution of enhanced green fluorescent protein (GFP) expression following AAV^PHP^ injection. (A)**Thirty days following intravenous injection of AAV^PHP^-eGFP, eGFP reporter expression was evident throughout the brain including in cortical and subcortical structures. **(B)** Immunofluorescent triple-labeling demonstrated that GFP reporter expression was restricted to GFAP-positive astrocytes but was not observed in NeuN-positive neurons. (**C** & **D**) Immunofluorescent labeling demonstrated that GFP reporter expression was not present in CD31-positive endothelial cells in AAV8-M1-GFP and AAV8-M23-GFP treated mice. Scale bar = 50 µm.


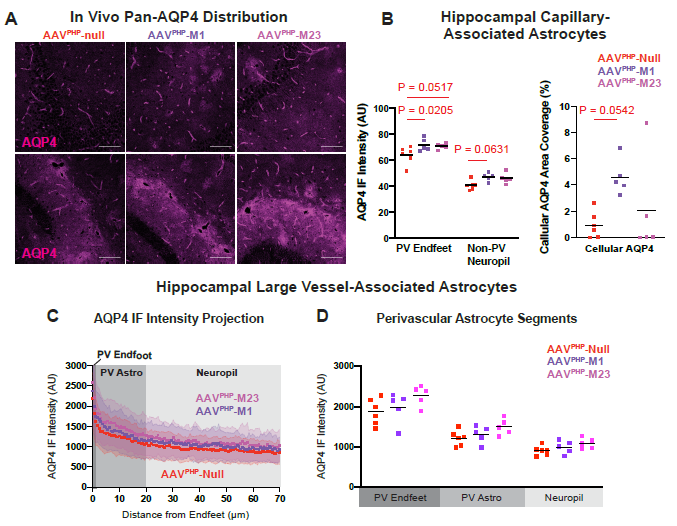


**Supplemental Figure 10. Overexpression of untagged AQP4-M1 and AQP4-M23 in mouse hippocampus.** (**A**) Thirty days after the viral delivery of untagged AQP4-M1 and -M23 isoforms, hippocampal AQP4 expression along capillaries (top) and large vessels (bottom) was evaluated using a pan-AQP4 antibody and confocal imaging. Scale bar: 100 µm. (**B**) In hippocampal capillary-associated astrocytes, overexpression of AQP4-M1 isoform resulted in a significant increase of the AQP4 IF intensity at the PV Endfeet (left; P = 0.0205, 2-way ANOVA with Tukey’s post hoc test). Overexpression of AQP4-M1 isoform also resulted in a numeric increase of the AQP4 IF intensity in non-perivascular neuropil (left; P = 0.0631, 2-way ANOVA with Tukey’s post hoc test) and cellular AQP4 coverage (right; P = 0.0542, 1-way ANOVA with Tukey’s post hoc test). Overexpression of AQP4-M23 isoform resulted in a numeric increase of AQP4 IF at the PV Endfeet (left; P = 0.0517, 2-way ANOVA with Tukey’s post hoc test). (**C-D**) Cross-sectional analysis of AQP4 IF at hippocampal large vessels showed that compared to animals treated with control virus (122 vessels from 6 animals), neither AQP4-M1 (81 vessels from 5 animals) or AQP4-M23 (85 vessels from 5 animals) overexpression resulted in changes in AQP4 IF in PV Endfoot, PV Astro or neuropil segments.


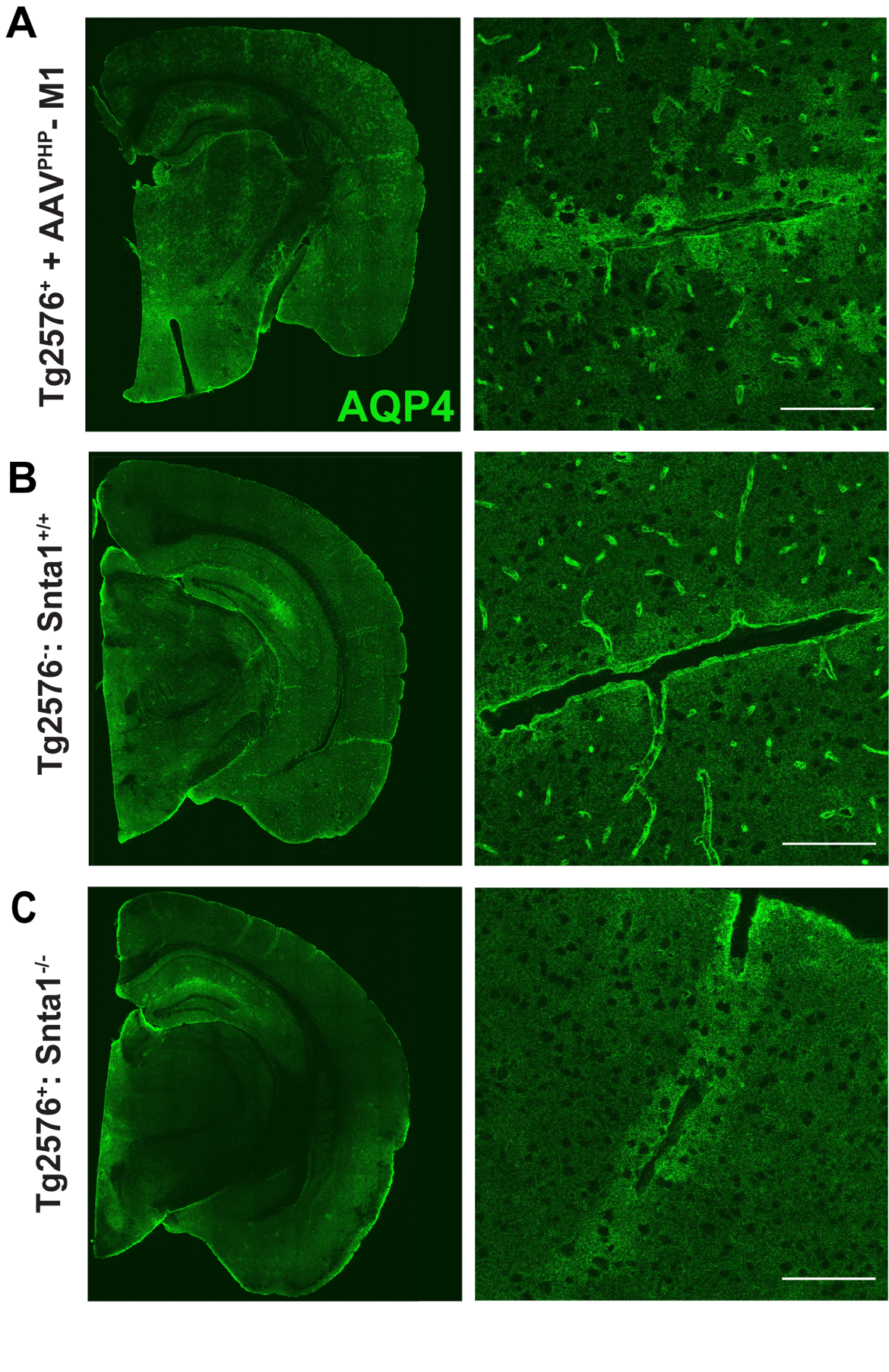


**Supplemental Figure 11: AQP4 localization at 6 months of age in the Tg2576 mouse brain following treatment with AAV-AQP4-M1 or genetic deletion of *Snta1*.** (A) Representative images of immunofluorescent labeling of AQP4 in 6 month old Tg2576 mice 3 months after injection with AAV^PHP^-M1. Overexpression of AQP4-M1 persisted in Tg2576 mice at 6 months of age. Representative images of immunofluorescent labeling of AQP4 are also shown for Tg2576^-^:*Snta1*^+/+^  **(B)** and Tg2576^+^:*Snta1*^-/-^ mice **(C)** at 6 months of age. Whole hemispheres are shown on the left, large penetrating vessels are shown on the right. Scale bar 100 µm.


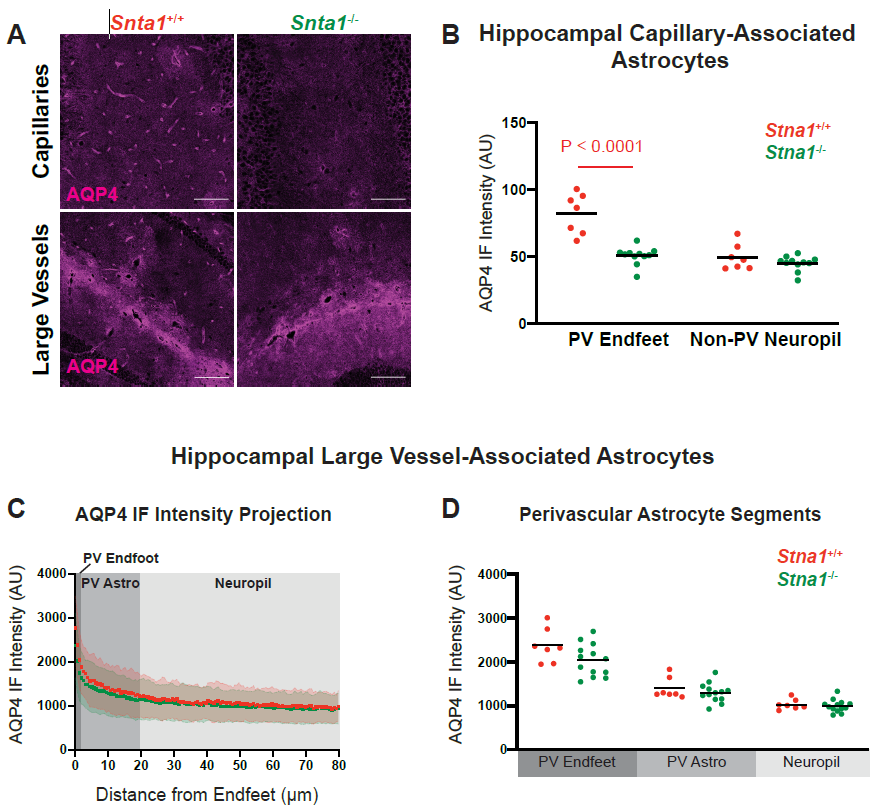


**Supplemental Figure 12. Hippocampal AQP4 localization in *Snta1*^-/-^ mice.** (**A**) Representative confocal images of the hippocampal AQP4 expression along capillaries (top) and large vessels (bottom) in wild type and *Snta1*^-/-^ mice. Scale bar: 100 µm. (**B**) In hippocampal capillary-associated astrocytes, PV Endfoot AQP4 IF was reduced to the levels in the non-perivascular neuropil in *Snta1*^-/-^ mice (P<0.0001, Mixed effects model with Sidak’s post hoc test), while the neuropil AQP4 IF was not altered. (**C-D**) Cross-sectional analysis of AQP4 IF associated with large hippocampal vessels showed that AQP4 IF did not significantly differ between *Snta1*^+/+^ mice (119 vessels from 7 animals) and *Snta1*^-/-^ mice (226 vessels from 13 animals) in the PV Endfoot, PV Astro or neuropil segments.


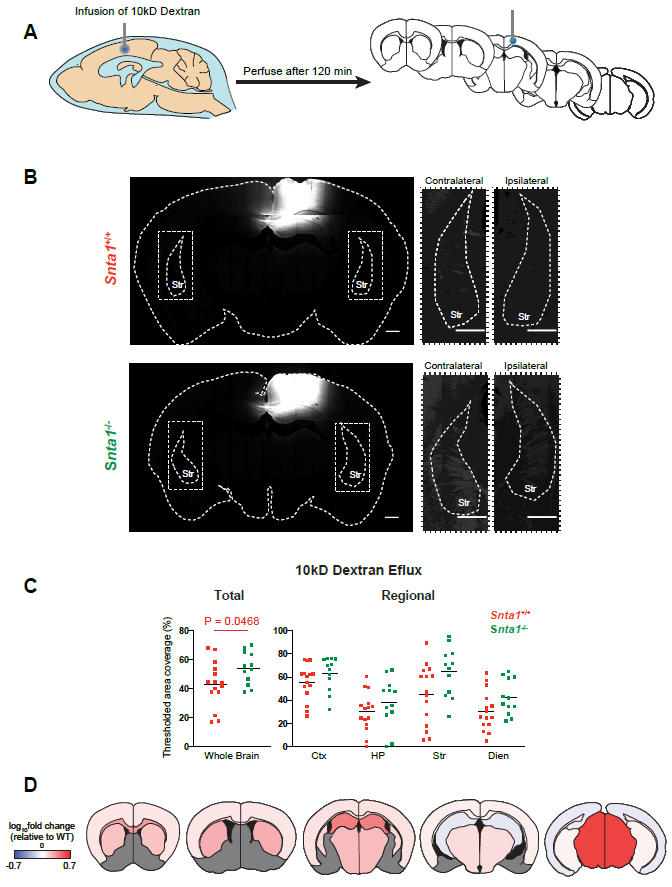


**Supplemental Figure 13. Interstitial solute efflux is reduced in the *Snta1*^-/-^ brain.** (**A**) Schematic outline of the intraparenchymal tracer injection followed by tracer visualization. Cascade Blue-conjugated dextran (10 kD) was intraparenchymally injected into the motor cortex of *Snta1*^-/-^ and wild type mice. Two hours after the injection, brains were perfusion-fixed and the interstitial tracer distribution across brain regions was evaluated in under whole-slice fluorescence microscopy. Distribution was defined by area coverage of tracer fluorescence within the ipsilateral and contralateral cortex, hippocampus, striatum and diencephalon integrated through five standard coronal slices per animal (2.5, 1.5, 0, -1 and -2 mm relative to bregma). (**B**): Representative images of the tracer distribution 120 minutes after the intraparenchymal injection in wild type and *Snta1*^-/-^ forebrain (left panel). Contralateral and ipsilateral striatal regions were zoomed in for better visualization (right panel). (**C**) The global tracer fluorescence intensity across all slices was significantly higher in *Snta1*^-/-^ mice compared to the wild type animals (P=0.0468, unpaired t-test; P=0.0340, Mixed effect model with Sidak’s post hoc test). Schematized heat map depicting regions with greatest differences in tracer distribution between *Snta1^-/-^* and *Snta1^+/+^* mice shows greatest retention of interstitial solutes in deeper, posterior brain structures.


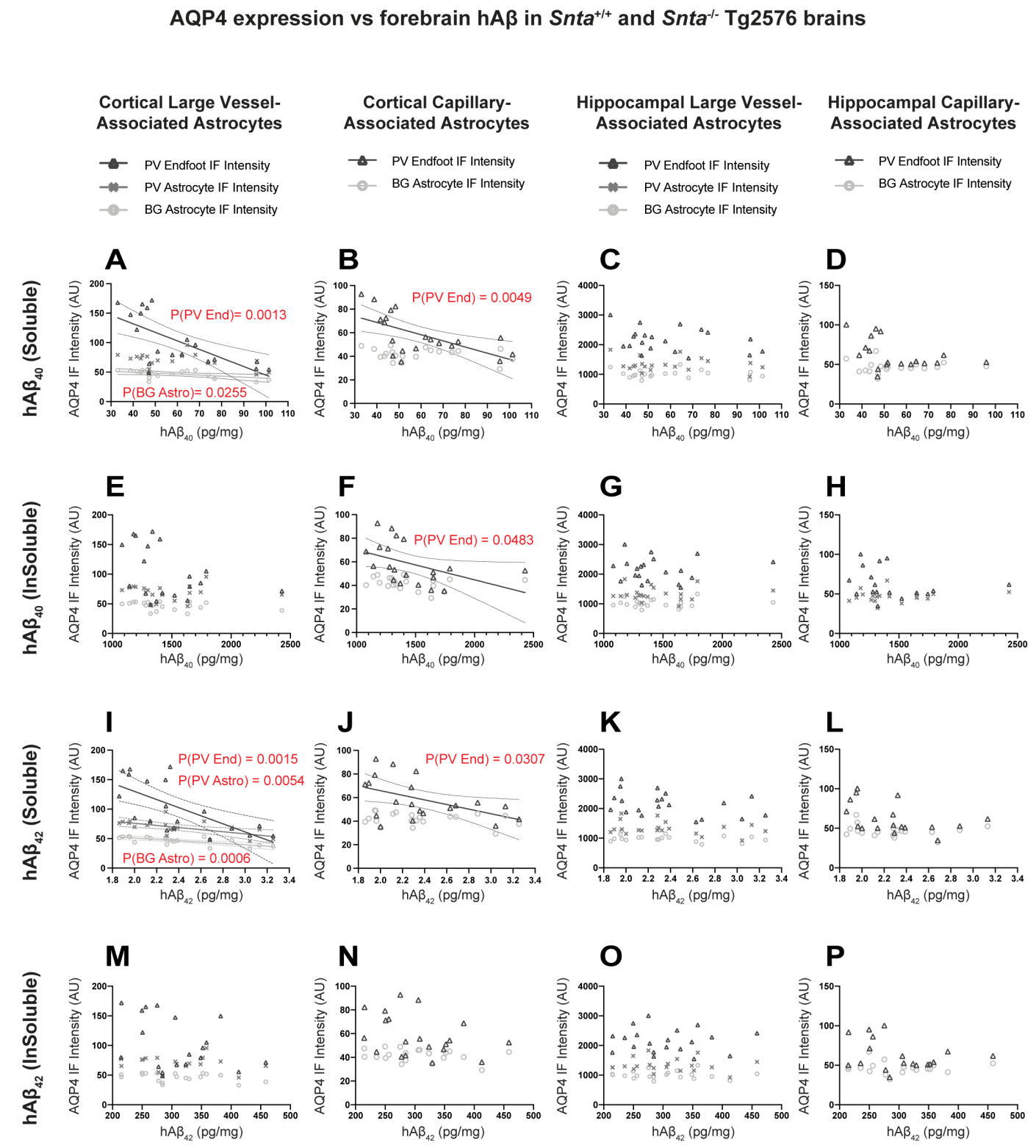


**Supplemental Figure 14. Correlation between segmental AQP4 IF and forebrain Aβ in Tg2576 mice with or without *Snta1* gene expression.** (**A-D**) Soluble Aβ_40_, (**E-H**) insoluble Aβ_40_, (**I-L**) soluble Aβ_42_, (**M-O**) insoluble Aβ_42_ concentrations were plotted against cortical and hippocampal large vessel- and capillary-associated segmental AQP4 IF values measured from the same animals. Soluble Aβ_40_ was negatively associated large vessel PV Endfoot (P_PV-endfeet_ = 0.0013, R^2^_PV-endfeet_ = 0.4651), non-perivascular neuropil (P_Neuropil_ = 0.0255, R^2^_Neuropil_ = 0.2606) AQP4 IF, and capillary PV Endfoot (P_PV-endfeet_ = 0.0049, R^2^_PV-endfeet_ = 0.3640) AQP4 IF. Insoluble Aβ_40_ was negatively associated capillary PV Endfoot (P_PV-endfeet_ = 0.0483, R^2^_PV-endfeet_ = 0.1996) AQP4 IF. Soluble Aβ_42_ was negatively associated large vessel PV Endfoot (P_PV-endfeet_ = 0.0015, R^2^_PV-endfeet_ = 0.4574), PV Astro (P_PV-astrocyte_ = 0.0054, R^2^_PV-astrocyte_ = 0.3742) and non-perivascular neuropil (P_Neuropil_ = 0.0005, R^2^_Neuropil_ = 0.5180) AQP4 IF, and capillary PV Endfoot (P_PV-endfeet_ = 0.0307, R^2^_PV-endfeet_ = 0.2340) AQP4 IF. No associations were observed between hippocampal AQP4 IF and Aβ levels of any species.


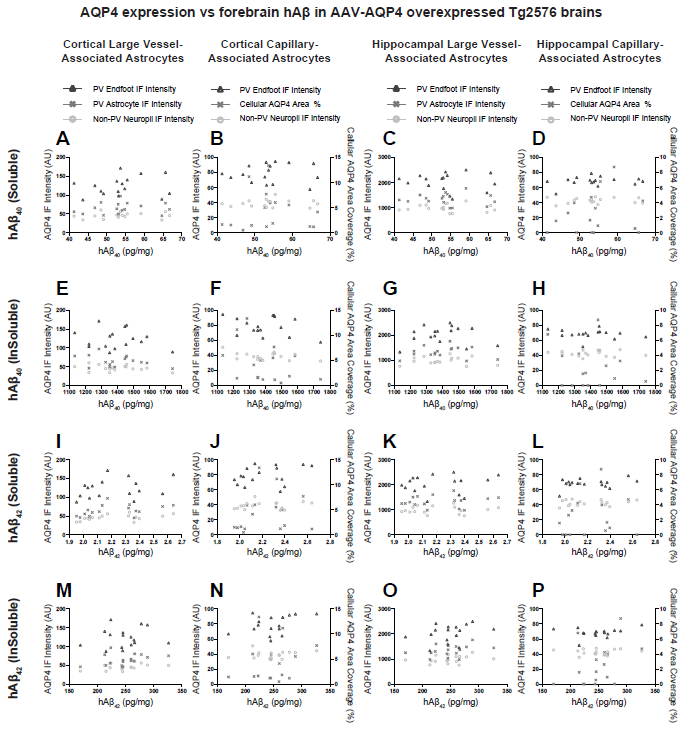


**Supplemental Figure 15. No correlation between the segmental AQP4 IF and forebrain Aβ in AAV-treated Tg2576 mice. (A-D**) Soluble Aβ_40_, (**E-H**) insoluble Aβ_40_, (**I-L**) soluble Aβ_42_, (**M-O**) insoluble Aβ_42_ concentrations were plotted against cortical and hippocampal large vessel- and capillary-associated segmental AQP4 IF values measured from the same animals. No significant associations were observed between large vessel- or capillary-associated AQP4 IF and Aβ burden among Tg2576 animals treated with AAV^PHP^-M1, AAV^PHP^-M23, or AAV^PHP^-null vector.
